# A transcriptome multi-tissue analysis identifies biological pathways and genes associated with variations in feed efficiency of growing pigs

**DOI:** 10.1186/s12864-017-3639-0

**Published:** 2017-03-21

**Authors:** Florence Gondret, Annie Vincent, Magalie Houée-Bigot, Anne Siegel, Sandrine Lagarrigue, David Causeur, Hélène Gilbert, Isabelle Louveau

**Affiliations:** 1 Pegase, Agrocampus Ouest, INRA, 35590 Saint-Gilles, France; 2 Laboratoire de Mathématiques Appliquées, IRMAR, Agrocampus Ouest, 35000 Rennes, France; 3 IRISA, CNRS, Université Rennes-1, INRIA, 35042 Rennes cedex, France; 4 GenPhySE, INRA, ENVT, Université de Toulouse, 31326 Castanet-Tolosan cedex, France

**Keywords:** Feed efficiency, Multiple factor analysis, Multi-tissues, Pig, Residual feed intake, Transcriptome

## Abstract

**Background:**

Animal’s efficiency in converting feed into lean gain is a critical issue for the profitability of meat industries. This study aimed to describe shared and specific molecular responses in different tissues of pigs divergently selected over eight generations for residual feed intake (RFI).

**Results:**

Pigs from the low RFI line had an improved gain-to-feed ratio during the test period and displayed higher leanness but similar adiposity when compared with pigs from the high RFI line at 132 days of age. Transcriptomics data were generated from *longissimus* muscle, liver and two adipose tissues using a porcine microarray and analyzed for the line effect (*n* = 24 pigs per line). The most apparent effect of the line was seen in muscle, whereas subcutaneous adipose tissue was the less affected tissue. Molecular data were analyzed by bioinformatics and subjected to multidimensional statistics to identify common biological processes across tissues and key genes participating to differences in the genetics of feed efficiency. Immune response, response to oxidative stress and protein metabolism were the main biological pathways shared by the four tissues that distinguished pigs from the low or high RFI lines. Many immune genes were under-expressed in the four tissues of the most efficient pigs. The main genes contributing to difference between pigs from the low vs high RFI lines were *CD40*, *CTSC* and *NTN1*. Different genes associated with energy use were modulated in a tissue-specific manner between the two lines. The gene expression program related to glycogen utilization was specifically up-regulated in muscle of pigs from the low RFI line (more efficient). Genes involved in fatty acid oxidation were down-regulated in muscle but were promoted in adipose tissues of the same pigs when compared with pigs from the high RFI line (less efficient). This underlined opposite line-associated strategies for energy use in skeletal muscle and adipose tissue. Genes related to cholesterol synthesis and efflux in liver and perirenal fat were also differentially regulated in pigs from the low vs high RFI lines.

**Conclusions:**

Non-productive functions such as immunity, defense against pathogens and oxidative stress contribute likely to inter-individual variations in feed efficiency.

**Electronic supplementary material:**

The online version of this article (doi:10.1186/s12864-017-3639-0) contains supplementary material, which is available to authorized users.

## Background

Feed cost is the major component (two-third or more) of the total cost of pig meat production [1], so that animal’s efficiency in converting feed into a rapid increase of lean body weight (BW) is a critical issue for the profitability of meat industries. To date, the main measures of feed efficiency in production farms are the feed conversion ratio (FCR) defined as the ratio of feed intake to the average daily gain (ADG) during a reference period, and its opposite trait, the gain-to-feed ratio (G:F). Both FCR and G:F are ratio traits and as such, can lead to unexpected responses on the component traits when improved through direct selection. In addition, Koch et al. [2] have defined the concept of residual feed intake (RFI) as the difference between animal’s actual feed intake and its predicted feed intake based on production needs. RFI is often regarded as a biological estimate of the efficiency of feed utilization, and the lower the RFI value is, the more efficient the animal is. Selection to improve feed efficiency through RFI has been proposed in different species. In pigs, lines divergently selected for RFI provided a good biological resource to unravel molecular mechanisms and functions associated with feed efficiency differences independent from the performance [3].

To date, some DNA variants playing a role in feed efficiency traits have been proposed from analyses of SNP locations by whole-genome association in pigs [4, 5] and beef cattle [6, 7]. Based on their genomic location in the vicinity of these SNPs, putative gene candidates for feed efficiency traits including RFI have been suggested. The highlighted genes pertain to numerous biological processes [8, 9], suggesting that biological strategies recruited for improving feed efficiency are diverse. To examine the variety of functional pathways underlying inter-individual difference in feed efficiency, an increasing number of studies have also addressed the transcriptomes and miRNA profiles of tissues and organs involved in energy homeostasis and energy demand in pigs [10, 11] and cattle [12–15] with contrasted RFI values. With the exception of Weber and colleagues [16] who very recently performed a multi-tissue transcriptome analysis in bulls, all other authors have addressed the different tissues separately. This did not allow understanding how shared pathways in different tissues and(or) tissue specific concerted pathways may participate to RFI and feed efficiency differences. Progress in this field is needed to predict side-effects of selection programs for RFI on unexpected animal phenotype and suggest other strategies to improve feed efficiency.

This study aimed to analyze the transcriptomic profiles of *longissimus* muscle, liver and two adipose tissues obtained in pigs from two lines divergently selected for RFI. Pigs from the low RFI line had an improved G:F ratio and they consistently demonstrated greater loin muscle size when compared with pigs from the high RFI line [17, 18]. This study focused on common *vs.* specific molecular pathways in different tissues which might be important in an efficiency phenotype.

## Results

### Growth and body composition of pigs from two lines divergently selected for RFI

Forty-eight pigs from two genetic lines (*n* = 24 per line) that were divergently selected over eight generations for RFI were compared at the same age (132 days). Pigs from the low RFI line grew faster during the test period (*P* < 0.001), ate somewhat less feed (*P* < 0.10) and were heavier at the same age than pigs from the high RFI line (Table 1). Gain-to-feed (G:F) ratio during the test period was clearly improved (*P* < 0.001) in pigs from the low RFI line. The *longissimus* muscle, the major fast-twitch muscle in the loin part of the body, had a greater mass (relative to BW) in pigs from the low RFI line, but liver was lighter in those pigs as compared with pigs from the high RFI line. The relative proportions of dorsal (backfat) subcutaneous (SCAT) and perirenal (PRAT, a fat depot surrounding kidneys) adipose tissues, which are used as surrogates of carcass adiposity, were almost similar in both lines.

**Table 1:** Growth and body composition traits in pigs from the low or high RFI lines

Line^*a*^	Low RFI	High RFI	*sem*	*P*-line
Pigs, n	24	24		
Age (d)	132	131	4	-
Body weight (kg)	77.8	73.3	5.5	0.008
Feed intake (g/d)	2250	2307	106	0.099
Average daily gain (g/d)	882	796	73	<0.001
Gain to feed (G:F) ratio, 77–132 d	0.38	0.35	0.02	<0.001
Dorsal subcutaneous AT thickness (mm)	13.6	14.0	1.03	0.49
Weight of tissues				
Liver (%BW)	2.11	2.32	0.19	<0.001
*Longissimus* muscle (%BW)	4.39	3.99	0.32	<0.001
Dorsal subcutaneous AT (%BW)	4.79	5.00	0.78	0.34
Perirenal AT (%BW)	0.70	0.69	0.12	0.51

^a^Two pig lines were divergently selected over eight generations for residual feed intake (RFI). A subset of 48 pigs was tested for performance from 74 days to 132 days of age and used tissue sampling. The resulting phenotype in this subset was an improved gain-to-feed ratio and greater muscularity in pigs from the low RFI line when compared with pigs from the high RFI line. *AT* Adipose tissue, *BW* Body weight

### Tissue transcriptomes

**Fig 1:**
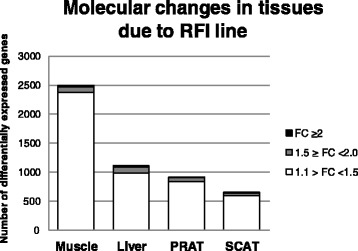
Molecular changes in tissues due to RFI line. Microarray data obtained in *longissimus* muscle, liver, perirenal (PRAT) and subcutaneous (SCAT) adipose tissues were separately analyzed for the main effects of line (low RFI: residual feed intake below the average; high RFI: residual feed intake above the average). Genes were declared as differentially expressed (DEG) between pigs from the low or high RFI lines according to the same cutoffs in the four tissues (*P* < 0.01 and FC between conditions > |1.1|). FC: fold-change between mean values calculated in pigs from the low or high RFI lines. Values are inversed and preceeded by a minus sign for FC < 1 (e.g., FC = 0.5 was indicated as FC = −2)

Microarray analyses were performed in *longissimus* muscle, liver, SCAT and PRAT of pigs from the low vs high RFI lines (*n* = 24 per line). By using the same cut-offs for the four tissues examined, the global effects of the line on the numbers of differentially expressed probes (DEP) and their corresponding genes (DEG) were found much more pronounced in muscle than in the other examined tissues, with a total of 2 494 genes being differentially-expressed in muscle (Fig 1). This number was more than two-fold lower in liver and PRAT and was about four-fold lower in SCAT. Most of DEG showed a fold-change less than |1.5| in pigs from the low vs high RFI lines.

**Fig 2:**
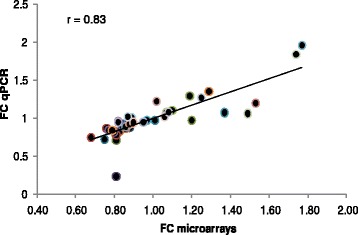
Reliability of microarray data. A subset of genes was analyzed by qPCR in *longissimus* muscle, liver, perirenal adipose tissue and subcutaneous adipose tissue (*n* = 48 in each tissue). The fold-changes (FC) calculated between expression levels of target genes in pigs from the low to high RFI lines in microarrays on one hand and qPCR on the other hand, were plot together. Correlation coefficient was calculated between the two measures. Forward and reverse primers used for qPCR are listed in Additional file 3

Altogether, more than one-half of the DEG (50% in liver and PRAT, and 60% in muscle and SCAT) were over-expressed in pigs from the low RFI line when compared with pigs from the high RFI line (Additional file 1). The lists of DEG are provided tissue by tissue in Additional file 2. The quality and reliability of the microarray results was checked by measuring the expression of target genes by qPCR from 6 (SCAT) to 11 genes (muscle, liver and PRAT) in each tissue (Additional file 3). A good correlation (r = 0.83) was generally found between microarrays and qPCR data (Fig. 2).

### Shared biological processes and gene networks across tissues targeted by RFI selection

**Fig 3:**
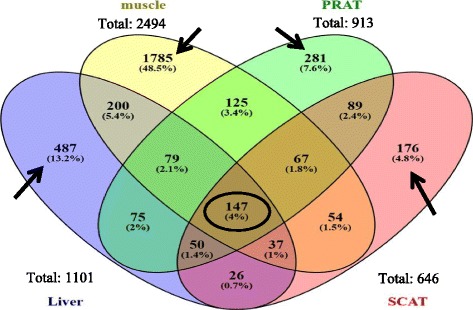
VENN diagram representing DEG due to RFI line. The lists of differentially expressed genes (DEG) due to line effect in *longissimus* muscle, liver, perirenal (PRAT) and subcutaneous (SCAT) adipose tissues were considered to edit the VENN diagram, using the VENNY tool [http://bioinfogp.cnb.csic.es/tools/venny/index.html]. A total of 147 DEG were commonly found in the four tissues (black circle). Black arrows point to the DEG exclusively found in one tissue. RFI: residual feed intake

Among the DEG, 147 unique genes were identified by the VENN diagram as common to the four tissues, and a much greater number were rather tissue-specific (Fig. 3). Fold-change (FC) in values of the gene expressions (pigs of the low vs high RFI lines) which were common to the four examined tissues as well in specific genes exclusively found in one tissue are provided in Additional file 4.

The 147 DEG were submitted to an enrichment test to explore the biological processes which were common to the four tissues. As indicated in Table 2, eight main biological pathways of clustered genes (enrichment score E ≥ 1, *P* < 0.05) were affected by the line across the four tissues. Intracellular (protein) transport and immune response, which includes antigen processing and presentation, were the top-enriched biological processes. In addition, membrane lipid metabolic process, macromolecule (protein) catabolic process, translational elongation, phospholipid metabolic process, the response to oxidative stress, and an heterogeneous cluster related to cell death, cytokine production and protein kinase activity were also listed as important biological processes modulated due to line. These eight pathways can be thus considered as the main biological responses shared by different tissues and participating in difference in feed efficiency.

**Table 2:** Main commonly affected biological processes across four tissues

GO terms^a^	Nb DEG	E score	*p*-value	Clustered DEG
GO:0046907 ~ intracellular transport	17	2.06	<0.001	*PSEN1, YWHAZ, TOMM5, MLX, DST, RAB35, PIKFYVE, MYL6, PEX6, STAM2, OPTN, CDK5, CCHCR1, YWHAG, JAK2, STAM, NUP43*
(GO:0015031 ~ protein transport)	(13)		(0.025)	
GO:0006955 ~ immune response	13	1.81	0.050	*HLA-A, HLA-B, HLA-C, HLA-H, CTSC, YWHAZ, PSEN1, TRAF6, POLR3B, BST1, COL4A3BP, ADAM17, XCL1*
(GO:0048002 ~ antigen processing and presentation of antigen)	(4)		(0.001)	
GO:0006643 ~ membrane lipid metabolic process	4	1.43	0.030	*CLN3, PSAP, SGMS1, COL4A3BP*
GO:0009057 ~ macromolecule catabolic process	17	1.14	0.005	*PSEN1, YME1L1,CD40, TRAF6, USP33, USP30, UBE2V1, PSMA3, PSMD1, MUTYH, CLN3, TMEM189, CDK5, RNASET2, NEURL2, ADAM9, ADAM17*
(GO:0030163 ~ protein catabolic process)	(12)		(0.015)	
GO:0006414 ~ translational elongation	4	1.13	0.052	*RPL6, RPL14, RPS18, RPL35*
GO:0008219 ~ cell death	14	1.08	0.007	*PSEN1, NTN1, CD40, TRAF6, SOD2, YWHAG, POLR3D, HSPE1, CLN3, TRIO, SGMS1, OPTN, PDCD4, CDK5, JAK2, BLCAP, ADAM9, ADAM17*
GO:0045859 ~ regulation of protein kinase activity	9		0.008	
GO:0001819 ~ positive regulation of cytokine production	5		0.007	
GO:0006644 ~ phospholipid metabolic process	6	1.06	0.021	*PON1, PIGL, PLA2G12A, SGMS1, PIKFYVE, CLN3*
GO:0006979 ~ response to oxidative stress	6	1.00	0.048	*PSEN1, SOD2, GPX3, JAK2, ADAM9, MUTYH*

^a^The 147 differentially-expressed genes (DEG) (pigs from the low vs high RFI lines) which were commonly listed in muscle, liver, perirenal and subcutaneous adipose tissues, were clustered based on shared gene ontology (GO) terms for biological processes. The enrichment score (E ≥ 1) and *P*-value for each cluster are provided

**Fig 4:**
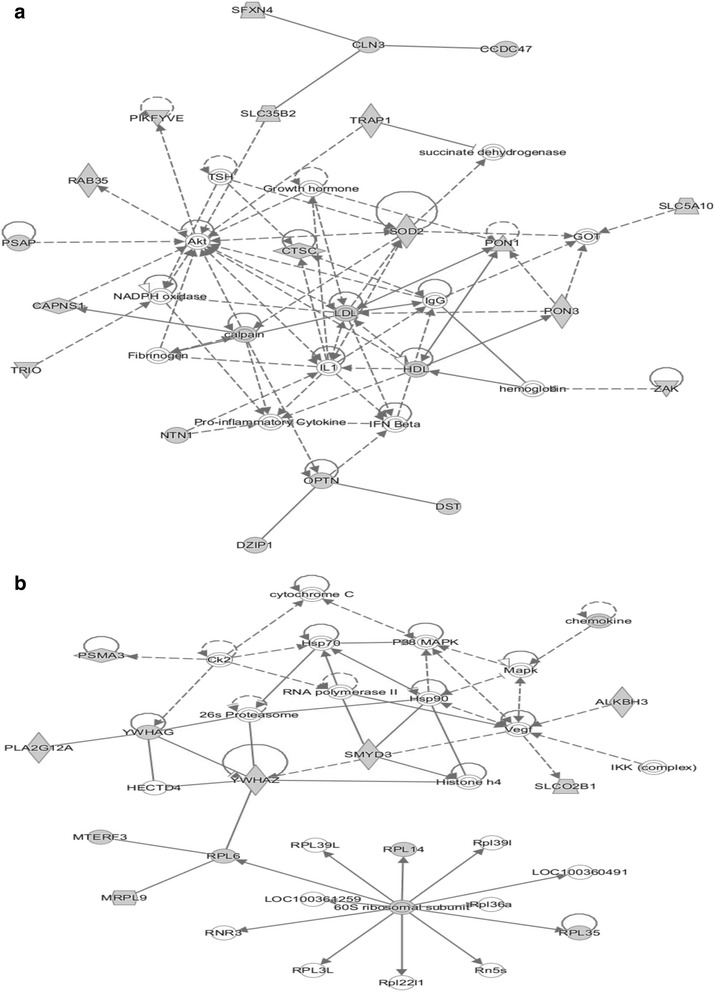
Examples of functional networks of regulated genes shared by different tissues. The 147 differentially expressed genes commonly listed in *longissimus* muscle, liver, perirenal and subcutaneous adipose tissues were submitted to Ingenuity® Pathway Analysis to visualize small networks of co-expressed genes. Highly-interconnected non-redundant networks were released from specified genes (represented as nodes) and relationships (represented as edges) between them or with neighboring genes were established based on literature records in the Ingenuity® Pathway Knowledge Base. This allows identifying functional networks related to response to oxidative stress, apoptosis, immunity and(or) protein metabolism. Two small functional networks are shown. More networks can be viewed in Additional files 5 and 6. **a** Relationships through low density lipoproteins (LDL), between *SOD2* and *PON1* (response to oxidative stress), *IL1* and *CTSC* (immunity) and *NTN1* (apoptosis). **b** Relationship between different molecules acting in protein translation (*RPL6, RPL14*), protein catabolism (*PSMA3*, 26S proteasome, and heat-shock proteins HSP70) and protein transport (*YWHAZ*)

In parallel, highly-interconnected non-redundant small networks were released using Ingenuity Pathway Analysis® (IPA) tool, with particular genes (out of the 147 DEG) represented as nodes and relationships between them or with neighboring genes represented as edges. This analysis allowed pointing key genes in different functional networks which were mainly related to response to oxidative stress, apoptosis, immunity and(or) protein metabolism. Through low density lipoproteins (LDL), the first network (Fig. 4a) describes relationships between *SOD2* and *PON1* (involved in the response to oxidative stress), *IL1* and *CTSC* (acting in immunity) and *NTN1* (regulating apoptosis). Two additional networks were also related to immunity (Additional file 5): three members of the major histocompatibility complex family (*HLA-A/B/C*) were involved in a network of genes including the phosphoinositide protein kinase *(PDK1*)*,* an enzyme participating in lymphocyte homeostasis and function [19]; two genes of the TNF family (*CD40*, *TRAF6*) were associated with nuclear factor kappa-light-chain-enhancer of activated B cells (NFκB) and C-jun N-terminal kinase (JNK) signaling pathways in another small network. Another significant network was clearly dedicated to protein metabolism (Fig. 4b) with different molecules acting in protein translation (*RPL6, RPL14*), protein catabolism (*PSMA3*, 26S proteasome and heat-shock proteins HSP70), and protein transport (*YWHAZ*).

A last informative network (Additional file 6) described the phosphoinositide 3-kinase (PI3K) and ERK signaling pathways with possible connections with *CREB1* (cAMP responsive element binding protein 1), a gene known to induce transcription of genes involved in fatty acid metabolism. This network also included *YME1L1* (YME1 like 1 ATPase), a gene involved in mitochondrial protein metabolism, and *PSEN1* (prenesilin-1), a gene encoding a protein that has been proposed to be the catalytic component of the gamma-secretase protein complex involved in development and cell regulatory processes.

### Multiple factor statistical analysis to highlight important genes in transcriptomic variability

**Fig 5:**
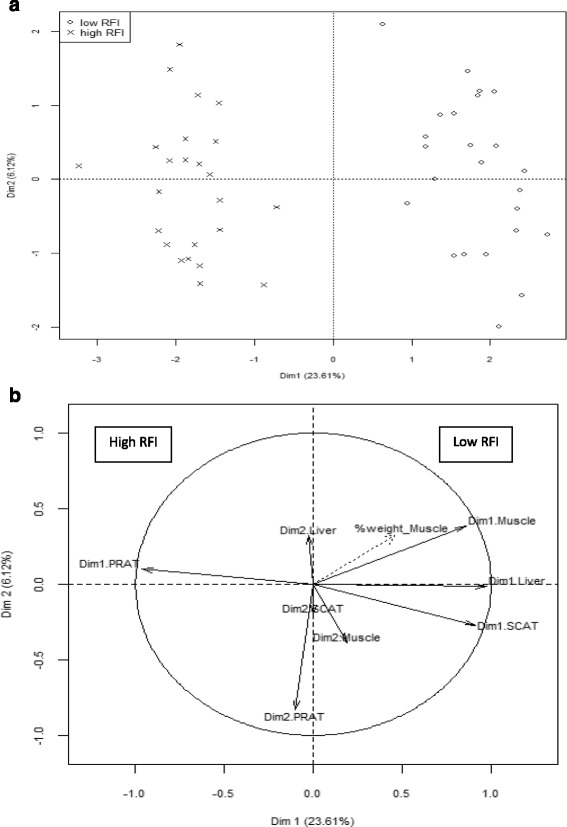
Multi-way mathematical datasets analysis: consensus between distinct tissues transcriptomes underlying separation of molecular data due to RFI line. **a** Pigs were represented on the scatter plot created with the first two dimensions (Dim) of the multiple factor analysis (MFA) which aggregated the whole transcriptomic variability across four tissues. Only the first dimension of the MFA (Dim1; 24% of the whole transcriptomic variability) discriminated pigs from the low RFI line and pigs from the high RFI line. **b** Synthetic latent variables were calculated from correlated molecular probes in the *longissimus* muscle (Dim 1_LL), liver (Dim 1_liver), perirenal adipose tissue (Dim 1_PRAT) and dorsal subcutaneous adipose tissue (Dim_1 SCAT) and projected in the correlation circle of MFA. The first latent variables in muscle (Dim1_muscle), liver (Dim1_Liver), perirenal adipose tissue (Dim1_PRAT) and subcutaneous adipose tissue (Dim1_SCAT) had a contribution near |1| to Dim1 in the diagnostic plot. This allows considering Dim1 of the MFA as a relevant summary of the main common molecular responses across the four tissues

In addition to bioinformatics analyses, a Multiple Factor Analysis (MFA) was performed to aggregate the whole transcriptomic datasets. This data integration mathematical procedure ensures that each tissue influence was balanced in the joint analysis when latent synthetic variables were calculated as linear combinations of the most highly correlated DEP. The first dimension (Dim1) of the MFA summarized 24% of the whole molecular variability. As shown in Fig. 5a, Dim1 clearly opposed pigs of the low RFI line (positive values) and pigs of the high RFI line (negative values). The muscle weight (%weight_muscle) was moderately correlated with Dim1 (*r* = 0.45, *P* = 0.001), which means that part of the molecular mechanisms summarized by Dim1 contributed to inter-individual difference in muscularity. The second dimension (Dim2) summarized only 6% of the variability of transcriptomic changes and this did not allow any distinction between pigs from the low or high RFI lines. Therefore, only Dim1 was further considered as a relevant summary of the tissue molecular changes due to line.

The first latent variables in muscle (Dim1_muscle), liver (Dim1_Liver), PRAT (Dim1_PRAT) and SCAT (Dim1_SCAT) had a contribution near |1| to Dim1 in the MFA diagnostic plot (Fig. 5b): this means that Dim1 of the MFA can be regarded as a relevant summary of similarities in the four tissues. The genes encoded by DEP having the highest correlation with Dim1 (r > |0.70|; *P* < 0.001) and commonly found in the four tissues are provided in Table 3. More detailed information can be found in Additional file 7.

**Table 3:** Top-ranked genes contributing to the first dimension of MFA analysis

Gene^a^	Full name (*Associated biological process)*	r
*PSEN1*	Presenilin 1 *(T cell receptor signaling pathway; regulation of MAP kinase activity)*	0.84
*PCIF1*	PDX1 C-terminal inhibiting factor 1 *(Negative regulation of phosphatase activity)*	0.77
*RPL6*	Ribosomal protein L6 *(Translation)*	0.77
*IPP*	Intracisternal A particle-promoted polypeptide *(Ubiquitination)*	0.77
*RPL14*	Ribosomal protein L14 *(Translation)*	0.76
*PHYH*	Phytanoyl-CoA 2-hydroxylase *(Fatty acid oxidation)*	0.76
*PLA2G12A*	Phospholipase A2 group XIIA *(Lipid catabolic process)*	0.75
*HEATR4*	HEAT repeat containing 4	0.75
*ZNF174*	Zinc finger protein 174 *(Transcription)*	0.74
*USP33*	Ubiquitin specific peptidase 33 *(Ubiquitin-dependent protein process)*	0.74
*CD40*	CD40 molecule *(Immune response)*	−0.79
*NTN1*	Netrin 1 *(Apoptotic process)*	−0.78
*POLR3H*	Polymerase (RNA) III subunit H *(Immune response)*	−0.77
*LZTFL1*	Leucine zipper transcription factor like 1 *(Protein localization)*	−0.76
*CYB561A3*	Cytochrome b561 family member A3 *(Oxido-reduction)*	−0.75
*H2-Q4*	Histocompatibility 2, Q region locus 4 *(Antigen processing)*	−0.75
*CTSC*	Cathepsin C *(Immune response)*	−0.74
*UQCRB*	Ubiquinol-cytochrome c reductase binding protein *(Oxido-reduction)*	−0.74
*PIKFYVE*	Phosphoinositide kinase, FYVE-type zinc finger containing *(Signal transduction)*	−0.74
*DST*	Dystonin *(Cytoskeleton organization)*	−0.74
*PHYH*	Phytanoyl-CoA 2-hydroxylase *(Fatty acid oxidation)*	−0.72

^a^Top-ranked contributors in transcriptomics variability were identified by a multiple factor analysis (MFA). Unique genes were ranked according to the correlation coefficient (r) calculated between their corresponding molecular probe(s) and the first dimension of the MFA that opposed pigs from the low RFI line to pigs from the high RFI line. When different probes corresponded to the same unique gene, only the highest r value for positive correlation or the lowest r value for negative correlation, respectively, is indicated. The main biological process associated to each gene in Entrez database was also indicated. *RFI* Residual feed intake

Among the top-ranked common genes, *PSEN1* (presenilin-1) was again identified; it had the strongest positive correlation with Dim1. Moreover, *USP33* (ubiquitin specific peptidase 33) and *IPP* (intracisternal A particle-promoted polypeptide) related to protein ubiquitination, *RPL6* and *RPL14* acting in translation, and *PLA2G12A* and *PIGL* involved in phospholipid metabolic process, also had positive contributions to Dim1. On the opposite, four genes involved in immune response (*CD40*, *CTSC, POLR3H* and *H2-Q4*) were identified as negative contributors to Dim1. Netrin (*NTN1*) and two other genes participating to oxido-reduction process (*CYBASC3, UQCRB*) were also among the top negative contributors to Dim1. Finally, *PHYH* (phytanoyl-CoA 2-hydroxylase) was represented by different DEPs being either positively or negatively associated to Dim1. This was due to an opposite regulation of *PHYH* expression in adipose tissues (up-regulated in PRAT and SCAT in pigs from low vs high RFI lines) and lean tissues (down-regulated in muscle and liver in pigs from low vs high RFI lines).

### Tissue specificities in the molecular differences between the two RFI lines

In a second step of analysis, genes that were differentially expressed only in one tissue were submitted to an enrichment test to reveal tissue specificities. Functional clusters were listed, tissue by tissue, in Tables 4, 5 and 6. Details on genes included in each cluster are provided in Additional files 8, 9 and 10.

**Table 4:** Summary of the main muscle-specific biological processes

GO terms^a^	Nb DEG	E score	*P*-value
Over-expressed in pigs from the low RFI line			
GO:0006412 ~ translation	97	39.8	<0.001
GO:0022613 ~ ribonucleoprotein complex biogenesis	33	4.3	<0.001
GO:0003012 ~ muscle system contraction process	26	4.1	<0.001
GO:0006091 ~ generation of precursor metabolites and energy	31	3.1	<0.001
(GO:0006006 ~ glucose metabolic process)	(26)		(0.007)
GO:0032268 ~ regulation of cellular protein metabolic process	52	2.8	0.001
(GO:0006417 ~ regulation of translation)	(23)		(<0.001)
GO:0030163 ~ protein catabolic process	67	2.7	<0.001
(GO:0006511 ~ ubiquitin-dependent catabolic process)	(28)		(0.010)
GO:0008104 ~ protein localization	84	2.7	0.003
(GO:0046907 ~ intracellular transport)	(73)		(<0.001)
GO:0005977 ~ glycogen metabolic process	8	1.6	0.009
GO:0046324 ~ regulation of glucose import	10	1.5	<0.001
Under-expressed in pigs from the low RFI line			
GO:0006955 ~ immune response	62	10.1	<0.001
GO:0002250 ~ adaptive immune response	18	5.4	<0.001
GO:0001817 ~ regulation of cytokine production	22	4.6	<0.001
GO:0010033 ~ response to organic substance	61	4.0	<0.001
GO:0070482 ~ response to oxygen levels	17	4.0	<0.001
GO:0001501 ~ skeletal system development	25	3.4	<0.001
GO:0042981 ~ regulation of apoptosis	55	3.0	<0.001
GO:0009743 ~ response to carbohydrate stimulus	10	2.1	<0.001
GO:0006631 ~ fatty acid metabolic process	27	1.7	0.006
(GO:0008610 ~ lipid biosynthetic process)	(19)		(0.05)

^a^Genes that were differentially expressed between pigs from the low or high RFI lines in muscle only were clustered according to gene ontology (GO) terms for biological processes and Genomes biological pathways (hsa). Significantly-enriched clusters (E ≥ 1; *P* < 0.05) were listed with the enrichment (E) score, the number (Nb) of differentially expressed genes (DEG) in each cluster and *P*-value. Overlapping clusters with a similar biological significance were removed

**Table 5:** Summary of the main liver-specific biological processes

GO terms^a^	Nb DEG	E score	*P*-value
Over-expressed in pigs from the low RFI line			
GO:0009100 ~ glycoprotein metabolic process	9	1.4	0.012
GO:0043691 ~ reverse cholesterol transport	3	1.0	0.024
GO:0007242 ~ intracellular signaling cascade	28	1.0	0.042
Under-expressed in pigs from the low RFI line			
GO:0006952 ~ defense response	17	1.9	0.013
hsa00591:Linoleic acid metabolism	5	1.4	0.001
GO:0042981 ~ regulation of apoptosis	19	1.4	0.034
GO:0033559 ~ unsaturated fatty acid metabolic process	4	1.3	0.035
GO:0006468 ~ protein amino acid phosphorylation	16	1.3	0.049
GO:0050778 ~ positive regulation of immune response	7	1.1	0.016
GO:0006959 ~ humoral immune response	6	1.0	0.005

^a^Genes that were differentially expressed between pigs from the low or high RFI lines in liver only were clustered according to considered to gene ontology (GO) terms for biological processes and Genomes biological pathways (hsa). Significantly-enriched clusters (E ≥ 1; *P* < 0.05) were listed with the enrichment (E) score, the number (Nb) of differentially expressed genes (DEG) in each cluster and *P*-value

**Table 6:** Summary of the main adipose tissue-specific biological processes

GO terms^a^	Nb DEG	E score	*P*-value
Subcutaneous adipose tissue			
Over-expressed in pigs from the low RFI line			
GO:0006796 ~ phosphate metabolic process	14	1.7	0.011
GO:0006006 ~ glucose metabolic process	5	1.6	0.018
GO:0006091 ~ generation of precursor metabolites and energy	11	1.5	0.004
(GO:0016042 ~ lipid catabolic process)	(6)		(0.006)
(GO:0006635 ~ fatty acid beta-oxidation)	(3)		(0.01)
GO:0034599 ~ cellular response to oxidative stress	4	1.2	0.033
Under-expressed in pigs from the low RFI line			
GO:0007517 ~ muscle organ development	6	1.7	0.002
GO:0006796 ~ phosphate metabolic process	9	1.3	0.045
GO:0006952 ~ defense response	7	1.2	0.041
Perirenal adipose tissue			
Under-expressed in pigs from the low RFI line			
GO:0001568 ~ blood vessel development	10	2.8	<0.001
GO:0008202 ~ steroid metabolic process	7	1.4	0.012
(GO:0008203 ~ cholesterol metabolic process)	(4)		(0.05)

^a^Genes that were differentially expressed between pigs from the low or high RFI lines in subcutaneous (SCAT) or perirenal (PRAT) adipose tissues only were clustered according to considered to gene ontology (GO) terms for biological processes and Genomes biological pathways (hsa). Significantly-enriched clusters (E ≥ 1) were listed with the enrichment (E) score, the number (Nb) of differentially expressed genes (DEG) in each cluster and *P*-value

#### Muscle

A total of 1 785 DEG were exclusively found in muscle (Fig. 3). This means that about 72% of muscle DEG was not listed in the other examined tissues. The highest number of up-regulated genes (97 DEG) in pigs from the low RFI line when compared with pigs from the high RFI line were participating to translation process (Table 4), with 60 genes coding for ribosomal subunits, 8 genes coding for mitochondrial ribosomes, 11 genes involved in translational initiation and 5 genes coding for translation elongation factors (Additional file 8). Intracellular transport and protein catabolic process, which included ubiquitin specific peptidases, ubiquitin-conjugated enzymes and proteasome subunits, were also top-enriched biological pathways of overexpressed muscular genes in pigs from the low RFI line. Another predominant pathway of over-expressed genes in muscle of pigs from the low RFI line was the contraction process with many up-regulated genes coding for filament proteins. Finally, over-expressed genes in muscle were also clustered in functional modules related to the generation of precursor metabolites and energy: they participated to the regulation of glucose import (~10 genes, including the insulin-related genes *INSR, INS-IGF2 and IRS2*), coded for various enzymes acting in glycogen metabolic process (~8 genes) and other glucose metabolic processes such as glycolysis, tricarboxylic acid cycle, and finally the respiratory chain (e.g.; cytochrome c oxidase-related and different NADH dehydrogenase subunits) to achieve the oxidative process of energy production from glucose (Additional file 8).

Conversely, 69 genes that were down-regulated in pigs from the low RFI line were involved in immune response and cytokine production (Table 4). They included genes associated with the Toll-like receptor family, complement components and receptors, and proteins regulating antigen presentation (Additional file 8). Under-expressed genes in those pigs also shared GOBP terms for fatty acid metabolic process, such as lipid biosynthetic process (19 genes). A high number of under-expressed genes in muscle of pigs from the low RFI line also participated to the regulation of apoptosis. Finally, skeletal muscle development, a process which was likely promoted by the transcription factor *MYC* and also included genes of the transforming growth factor (*TGFB1, TGFBR2*) and insulin-growth factor-1 (*IGF1, IGF1R*) families (Additional file 8), was identified as another down-regulated process in pigs from the low RFI line.

#### Liver

In liver, the majority of the up-regulated genes in pigs of the low RFI line compared with pigs of the high RFI line participated to intracellular signaling cascade (Table 5). Three overexpressed genes were also involved in the reverse cholesterol transport: *LIPC* (hepatic form of triglyceride lipase), *APOA1* (apolipoprotein-A1) coding for a major component of high-density lipoproteins, and *LCAT* (lecithin–cholesterol acyltransferase) which is responsible for the formation of plasma cholesteryl esters from free cholesterol (Additional file 9).

Most of the down-regulated genes in liver of pigs from the low RFI line compared with pigs from the high RFI line participated to defense and immune responses (Table 5). These pathways included TNF family members and interleukins, such as *IL10* (interleukin-10) produced by lymphocytes and its receptor (*IL10RB*), which is required for *IL10*-induced signal transduction (Additional file 9). Under-expressed hepatic genes in those pigs also participated to unsaturated fatty acid metabolism, with four members of the cytochrome P450 superfamily of enzymes known to catalyze reactions in the synthesis of cholesterol and steroids (Additional file 9).

#### Adipose tissues

The DEG in the two adipose tissues of pigs from the low RFI line compared with pigs from the high RFI line were distributed in a large number of biological processes, so that there were few (SCAT) or no (PRAT) significantly enriched pathways. Only 37% of the DEG in PRAT was also reported in SCAT (Fig. 3), which suggests different molecular responses according to fat anatomical locations.

In SCAT, the predominant biological pathway shared by over-expressed adipose genes in pigs from the low RFI line concerned the generation of precursor metabolites and energy. Notably, glucose metabolic process (5 genes) and lipid catabolic process (6 genes) were up-regulated processes in those pigs when compared with pigs from the high RFI line (Table 6); this latter pathway included *PLIN1* acting in lipolysis and three genes participating to fatty acid beta-oxidation (Additional file 10). The response to oxidative stress with genes encoding anti-oxidant enzymes (*CAT, SOD1, DUOX2*), was identified as another enriched biological process in SCAT of pigs from the low RFI line when compared with pigs from the high RFI line. Conversely, defense response was the main enriched pathway of under-expressed genes in SCAT in pigs from the low RFI line: this concerned chemokines (*CCL21, CCL8*) and attractin (*ATRN*), a secreted protein regulating the chemotactic activity.

Specific to PRAT, under-expressed genes in pigs from the low RFI line were participating to steroid and cholesterol metabolic processes (Table 6); they included members of the cytochrome P450 superfamily catalyzing reactions involved in synthesis of cholesterol, and *SREBF2* (sterol regulatory element binding transcription factor 2) acting as a key transcription factor to control cholesterol homeostasis (Additional file 10).

#### Conclusions on tissue specificities

Although represented by different genes in each tissue, immune and defense responses (muscle, liver and SCAT), regulation of apoptosis and cell death (muscle, liver), and cholesterol and steroid metabolic processes (liver, PRAT) thus emerged again as modulated pathways due to line. Importantly, fatty acid catabolic process was represented by genes that were under-expressed in muscle of pigs from the low RFI line and by genes that were up-regulated in adipose tissues of the same pigs when compared with pigs from the high RFI line.

## Discussion

This study considered pigs from two genetic lines divergently selected for RFI, a biological estimate of the efficiency of feed utilization. Phenotypically, pigs from the low RFI line had an improved gain-to-feed ratio during the test period, the difference between lines being commensurate to our previous observations in a former generation of selection [20]. In this experiment, this was achieved by increased growth rate rather than by large reduction in daily feed intake for pigs of the low RFI line when compared with pigs of the pig RFI line. Especially, the line-associated difference in feed intake in these subsets was smaller (57 g/d, *P* < 0.10) than what was expected based on data acquired in sib mates raised in the selection farm (240 g/d, personal data) and reported in former generations raised in experimental farms under conventional diets (90 g/d; [17; 18]). Pigs of the low RFI line also had greater muscle mass when compared with pigs from the high RFI line, which agrees with previous data [17, 18]. Altogether, it is important to state that the two groups inherited the intrinsic RFI genetic differences of their line; they were also phenotypically different in feed efficiency. Because it is now assumed that dedicated mathematical methods and joint modelling analyses are required to integrate multiple tissue transcript data [21], multivariate statistical analysis and descriptive bioinformatics were associated to propose a reasonable number of shared and tissue-specific functional pathways from muscle, liver and adipose tissues transcriptomes and to enlighten key genes which can be related to a genetic difference for RFI and a general difference in feed efficiency phenotype.

### Immune molecular pathways shared by the four tissues

Immune response appears to be the most important biological pathway of clustered under-expressed genes in the four tissues examined in pigs from the low RFI line (more efficient) when compared with pigs from the high RFI line (less efficient) at 132 days of age. In agreement with our results, levels of serum inflammatory markers such as the acute phase protein haptoglobin have been reported to be lower in Yorkshire pigs selected for low RFI [22]. In cattle, contrasted results have rather been reported. Consistent with our results, the expression levels of some genes related to inflammatory processes were lower in liver of low RFI heifers [23] and an enrichment of transcriptomic networks for inflammatory response was conversely reported in the liver of low feed efficient, high RFI steers [13]. On the opposite, pathway analysis of DEG across muscle, adipose tissue and duodenum predicted immune system and inflammatory response to be increased in low RFI bulls [16]. Because having an activated immune system is energetically costly [24], the down-regulation of many immune genes in pigs from the low RFI line (more efficient) may contribute to lower basal metabolic rate in those pigs. In support, a lower heat production related to reduced basal metabolic rate has been previously reported in low RFI pigs compared with high RFI pigs [20]. In our study, the over-expression of *CD40* (TNF receptor superfamily member 5) in the four examined tissues was notably proposed as one of the hallmarks of pigs from the high RFI line. This may be due to the regulatory role of this gene (which is expressed by T and B lymphocytes) in a wide spectrum of molecular processes during the initiation and progression of adaptive immunity [25, 26]. This gene has been also identified in a significant genomic region associated with ADG in pigs [9]. Another important gene in the inflammatory response to discriminate pigs from the low vs high RFI lines was *CTSC* (cathepsin-C). This gene codes for a lysosomal cysteine proteinase playing a central role in bacterial killing and immune regulation in T lymphocytes [27]. Identifying *NTN1* (netrin-1) as another key gene for which a differential expression in the four tissues brought an important contribution to genetics difference for RFI, may be related to the role of this laminin-related secreted protein in directing the retention of macrophages in adipose tissue [28]. In accordance, different genes coding for chemokines, a family of chemoattractant cytokines, were also identified with a lower expression in SCAT of pigs from the low RFI line. Instead, the transcriptional modulation of immunity in muscle was more specifically related to Toll-like receptors (*TLR2* and *TLR4*), which are known to be expressed by both myoblasts and myotubes [29]; genes in this pathway also included complement components and receptors that may initiate the inflammatory response in muscle [30]. From the current data, it is however not possible to predict the ability of pigs from the low or high RFI lines to cope with an immune challenge. In younger pigs, it has been shown that the two RFI lines coped similarly with a complete Freund’s adjuvant challenge inducing a noninfectious pneumonia [31]. Moreover, low RFI piglets recovered faster than high RFI piglets after the challenge [32]. In comparison with the high RFI line, growth rate was also less impaired in young growing pigs from the low RFI line facing degradation of hygiene conditions [33] and after a PRRSV infection [34]. These data suggest that having lower expression levels of various immune genes in tissues would not limit the ability of more efficient pigs to cope with inflammatory events in the young age; this remains to be checked for pigs around puberty.

### Anti-oxidant pathways in lean and fat tissues

The present study identifies response to oxidative stress as another biological process shared by the same genes which were differentially-regulated in the four tissues depending of the line. A small network of co-expressed genes also linked response to oxidative stress with immunity and apoptosis. In agreement with these findings, it is generally admitted that immune system activation releases highly reactive species to kill pathogens, which can cause oxidative damage to tissues. Especially, paraoxonase-1 (*PON1*) had a higher expression level in the four tissues of pigs from the low RFI line when compared with pigs from the high RFI line. This glycoprotein is transferred from liver to prevent the accumulation of lipid peroxides and reduce macrophage inflammatory response in tissues [35]. Conversely, an up-regulation in *UQCRB*, a gene coding for the ubiquinol-cytochrome c reductase binding protein which plays roles in oxygen sensing and ROS-mediated signaling [36], may negatively contribute to feed efficiency. Altogether, this suggests diminished oxidative stress in more efficient pigs. In agreement with this assumption, research in broilers has consistently shown that muscle mitochondria from high efficient birds produce less hydrogen peroxide [37]. In addition, others have shown that ROS production in muscle and RFI values were positively correlated, although no difference in electron leakage was seen in mitochondria prepared from livers of low and high RFI pig lines [38]. In our study, adipose-specific molecular responses to oxidative stress were further suggested, with the higher rather than lower expression levels in SCAT of different genes participating to the first enzymatic line of defense against oxidative stress (*CAT, SOD1, DUOX2*) when pigs from the low RFI line were compared with pigs from the high RFI line. This suggests that feed efficiency might be associated with different ROS production between adipose and lean tissues, and(or) that these tissues dealt differently with ROS detoxification.

### Up-regulation of genes involved in protein synthesis and catabolism in tissues

Finally, the largest number of genes differentially regulated in the four tissues between pigs from the low or high RFI lines belonged to protein metabolic processes. Similarly, candidate genes in pathways related to protein metabolism have been suggested in genomic regions accounting for feed conversion ratio in cattle [7]. Ribosome biogenesis and translation processes were notably enriched in over-expressed genes in tissues of pigs from the low RFI line. This could explain the greater muscle mass in those pigs when compared with pigs from the high RFI line at 132 days of age. Up-regulated genes acting in protein synthesis, proliferation and growth have been similarly revealed across muscle, liver and adipose tissue of low RFI beef cattle [12, 16]. Surprisingly, different genes known to participate to protein degradation were over-expressed in the four tissues examined in pigs from the low RFI line when compared with pigs of the high RFI line. This was not expected because others have reported depressed ubiquitin content in breast muscle of high feed efficient chicken [39] and lower activities of protein degradation enzymes in muscle of pigs selected for low RFI [40]. Among other genes involved in protein addressing and degradation, expression levels of *PSEN1* (presenilin-1) and *USP33* (ubiquitin specific peptidase 33) regulating ubiquitin-dependent protein catabolic process, were among the top molecular contributors in the differences between low and high RFI lines. Many ubiquitin-conjugating enzymes were also specifically over-expressed in muscle of the most efficient pigs. Both protein targeting and the ubiquitin system are known to regulate virtually all aspects of cellular functions [41]. Therefore, the observed changes in genes regulating protein transport and degradation may be important in the control of various biological processes involved in an improved efficiency phenotype. However, because protein synthesis, transport and breakdown are regarded as energy-costly processes [42], up-regulation of protein metabolism in tissues of pigs from the low RFI line represented an apparent paradox that deserves further studies.

### Tissue-specific modulations in energy use

Tight regulation of glucose homeostasis is essential for fast-twitch muscle where glucose metabolites are the major energy sources. In *longissimus* muscle, the gene expression program related to glycogen utilization was up-regulated in pigs from the low RFI line. This is in agreement with our previous findings of higher level of glucose issued from glycogen hydrolysis and elevated glycolytic potential in pigs selected for low RFI [17]. Moreover, increased glycogen content in hypertrophied white fast-twitch fibers have been previously observed in muscle biopsies from low RFI pigs in a former generation of selection [43]. In the present study, genes involved in the respiratory electron transport chain of mitochondria were also up-regulated in muscle of pigs from the low RFI line. This is in accordance with recent data showing higher mitochondrial protein expressions by shotgun proteomics in chicken with an improved feed efficiency phenotype [44]. Altogether, these data may sign for an increased efficiency of mitochondria rather than a greater number of mitochondria in the most efficient pigs. Conversely, different genes involved in triglyceride lipolysis, fatty acid oxidation but also fatty acid synthesis and elongation, were up-regulated in muscle of pigs from the high RFI line, which suggests that fatty acid use was promoted in muscle of the less efficient pigs. The greater activity of hydroxyacyl-Coenzyme A dehydrogenase, an important enzyme in the beta-oxidation, which was measured in muscle from high RFI pigs in a former generation of selection [18], further supports these molecular data. Because energy cost of intramuscular fatty acid synthesis is elevated, more efficient pigs may favor the use of glycogen rather than of fat to support energy needs for protein deposition and muscle filament contraction. Opposite to the muscle transcriptome, an increased expression of genes participating to lipolysis and fatty acid oxidation was observed in SCAT of pigs from the low RFI line. A striking example for this opposite regulation in fatty acid oxidation between lean (muscle and liver) and fat (adipose tissue) was provided by *PHYH.* This gene codes for the phytanoyl-CoA 2-hydroxylase, an enzyme involved in metabolizing phytanic acid, a metabolite of phytol regulating the alpha-oxidation process in the peroxisome. Examples for such an opposite regulation of *PHYH* gene expression between liver and adipose tissue have been provided in rodents fed high-fat diets [45, 46]. To date, a higher fatty oxidation in adipose tissue is regarded as a key for the lean phenotype in rodents and human [47]. The present study further suggests that fatty acid oxidation in adipose tissue could be important for the efficiency of lean growth, independently of adiposity.

Finally, members of the cytochrome P450 (*CYP*) superfamily of enzymes that catalyze reactions in the synthesis of cholesterol and steroids were down-regulated in liver and PRAT of pigs from the low RFI line (more efficient). Similarly to our results, other *CYP* genes were found as down-regulated genes in liver of low RFI cattle [12, 13]. Conversely, *LCAT* (lecithin cholesterol-acyltransferase) responsible for the formation of most plasma cholesteryl esters, and its cofactor *APOA1* (apolipoprotein A1) which promotes cholesterol efflux, were up-regulated in liver of pigs from the low RFI line. Taken together, these changes suggest that cholesterol level in liver may be reduced (less synthesis, greater efflux) in more efficient pigs. Interestingly, positive relationships between cholesterol plasma level and RFI values have been previously reported in pigs [48] and beef cattle [13]. Altogether, this suggests that cholesterol content may be associated with differences in efficiency phenotypes.

### Tissue hierarchy participating to an efficiency phenotype

Among the four examined tissues, the *longissimus* muscle was found as the primary site affected by a divergent selection for RFI. This was expected due to the main role of skeletal muscle in body energy homeostasis and energy use. Among genes involved in the regulation of glucose import, energy metabolic process and cellular protein metabolism, different genes related to insulin and *IGF2* system were over-expressed in muscle of pigs from the low RFI line, which is in agreement with recent findings showing an up-regulation of *IGF2* in muscle of more efficient pigs [49]. On the opposite, other genes known to participate to skeletal muscle development such as *c-MYC* antigen, *TGF-β*, *IGF1* and its receptor *IGF1R* were down-regulated in muscle of pigs from the low RFI line. Decreased circulating concentration of IGF-I has been also associated with a leaner, more efficient phenotype when juvenile pigs (range 33 d to 42 d of age) have been investigated [50]. Considering that *IGF1* expression in pig muscle decreased with advancing age [51], this suggests that difference in growth kinetics may exist between pigs from the low and high RFI lines. In the present study, dorsal subcutaneous adipose tissue (SCAT) had the less affected transcriptome among the four examined tissues. The selection procedure has been conducted with the objective to keep adiposity constant [3]. If any, phenotypic differences in subcutaneous adiposity between lines were not manifested before 19 weeks of age [17]. Altogether, time-course studies should be valuable to better understand the roles played by various biological mechanisms in muscle and adipose tissues in inter-individual differences in feed efficiency.

## Conclusion

This study presents novel findings on the transcriptomic modulations related to feed efficiency considering four tissues involved in energy generation and use in pigs. Immune response, protein metabolism and response to oxidative stress were identified as the main molecular pathways that were associated with a genetic difference for RFI and a general difference for efficiency phenotypes in pigs at 132 days of age. Therefore, non-productive functions in metabolic tissues are likely important processes for feed efficiency. It may be further suggested that dietary compounds with both anti-inflammatory and anti-oxidant activities can improve feed efficiency in growing pigs.

## Methods

### Ethic statement

The care and use of pigs were performed following the guidelines edited by the French Ministries of High Education and Research, and of Agriculture and Fisheries (http://ethique.ipbs.fr/sdv/charteexpeanimale.pdf). All pigs were reared in compliance with national regulations and according to procedures approved by the French Veterinary Services at INRA experimental facilities.

### Animals

Purebred Large White pigs were considered in the present study. These pigs originated from two lines divergently selected over eight generations for RFI. Principles of selection were described by Gilbert and colleagues [3]. Briefly, daily feed intake (DFI) was measured on group-housed boars fed ad libitum from 35 to 95 kg BW. Two traits were recorded and used to calculate the predicted feed intake: ADG from 35 to 95 kg BW and backfat thickness (BFT) at 95 kg BW assessed by an ultrasonic device and averaged from 6 different measures at the dorsal locations. A phenotypic RFI selection index was then computed as a linear combination of those traits as follows: RFI = DFI ‐ (1.06 × ADG) ‐ (37 × BFT). The objectives were keeping ADG and BFT constant between the two RFI lines.

In this experiment, 48 barrows (*n* = 24 per line) originated from the selection herd (INRA GenESI, Le Magneraud, France) were reared at INRA Pegase (St-Gilles, France). From 74 d of age onwards, pigs were randomized by line in two dietary groups (*n* = 12 per diet and per line), a high-fat high-fiber diet or a low-fat low-fiber diet. Detailed information about diets can be found in our associated publication [52]. All pigs were housed in isolated pens, thus allowing an individual control of DFI. At 132.5 d ± 0.5 d of age, the 48 pigs were killed in the post-prandial period (2 h after their last meal) by electronarcosis followed by jugular exsanguination. Samples were immediately collected at the dorsal subcutaneous adipose tissue (SCAT) location (last rib level) and from the *longissimus* muscle just below SCAT sampling in the right carcass side. After opening the abdominal cavity, the liver and the entire perirenal adipose tissue (PRAT) were collected and weighed. Tissue samples were immediately cut, frozen in liquid nitrogen, and stored at −75 °C until analyses. The day after slaughtering, the entire dorsal SCAT (backfat) and the *longissimus* muscle were removed from the left side of the chilled carcasses and weighed.

### RNA isolation and reverse transcription

Total RNA was extracted from each tissue by homogenization in Trizol reagent (Invitrogen, California, USA) and purified using a silica-membrane technology under vacuum (Nucleospin® 8 RNA kit, Macherey-Nagel, France). The method included a DNase digestion step to remove contaminating DNA. The quantification of RNA was performed by using a NanoDrop® ND-1000 spectrophotometer (Thermo Scientific, Illkirch, France). Ratios of A260/280 and A260/230 were greater than 1.7 in all samples, denoting good purity. The RNA integrity was assessed using the Agilent RNA 6000 Nano kit and an Agilent 2100 Bioanalyzer (Agilent Technologies France, Massy, France). Average RNA integrity numbers were 8.4 ± 0.4 (mean ± SD). Altogether, 192 RNA samples (*n* = 48 per tissue; 4 tissues) were thus generated for further analyses.

### RNA labelling and microarray hybridization

Custom porcine microarrays that contain 60,306 porcine probes were used (8 × 60 K, GPL16524, Agilent Technologies, Massy, France). Each of the 192 RNA samples was labeled with Cy3 dye using the Low Input Quick Amp Labeling kit (Agilent Technologies) following the manufacturer’s instructions. Briefly, a two-step procedure generates fluorescent complementary RNA (cRNA) by using T7 RNA polymerase, which simultaneously amplifies target and incorporates cyanine-labeled CTP. Samples were then purified with an RNeasy Mini kit (Qiagen, Hilden, Germany). Microarray hybridizations were carried out in Agilent’s hybridization chambers containing 600 ng of Cy3-labeled cRNA sample per hybridization. Hybridization reactions were performed at 65 °C for 17 h using Agilent’s Gene Expression Hybridization kit. After washing, microarrays were scanned at 3 μm/pixel resolution using the Agilent DNA Microarray Scanner, and images were analyzed with Agilent Feature Extraction Software (Version 10.7.3.1, protocol GE1_107_Sep09).

Analyses were performed using the R software version 2.10.0. Raw spots intensities were first submitted to quality filtration based on four criteria: intensity, uniformity, saturation and outliers detection. Intensities of filtered spots were transformed into log2(Cy3), and data were normalized by median centering, i.e. subtracting the median value across all probes from all raw values for each sample.

### Microarray data statistical analyses

Microarray consolidated data are deposited in NCBI’s Gene Expression Omnibus (GEO) and are accessible through GEO subseries accession numbers (http://www.ncbi.nlm.nih.gov/geo/query/acc.cgi?acc=) GSE84092 for *longissimus* muscle, GSE84093 for liver, GSE70837 for PRAT and GSE70836 for SCAT. First, the microarray data obtained in each tissue were analyzed by a two-way analysis of variance (ANOVA) with RFI line, diet and the interaction between line and diet as the main effects. Because there is no significant interaction between line and diet (cut-off *P* > 0.05), the line and diet effects can be separately inferred. In this study, only the line effect on transcriptomes was analyzed and discussed. For the diet effect on adipose tissue transcriptomes, one may refer to our associated publication [53]. The same cut-off (raw *P*-value < 0.01 and fold-change between conditions > |1.1|) was considered as a heuristic way [54] to retain a sufficient number DEP in the four tissues due to line allowing subsequent integrative analyses. Numbers of DEP when considering Benjamini-Hochberg (BH) multiplicity correction of the *P*-values for a control of the FDR were also calculated in the four tissues (Additional file 1). The lists of DEP (up-regulated vs. down-regulated in low RFI line compared with high RFI line) were generated by tissue, and the correspondence between each DEP and its official gene symbol was made to elicit lists of DEG.

To deduce communalities across the four tissues and to reveal molecular specificities of each tissue, the VENNY tool free online on the web site was used to compare the transcriptome data lists and edit VENN diagrams [http://bioinfogp.cnb.csic.es/tools/venny/index.html]. Because different molecular probes can correspond to the same unique gene, communalities and specificities were deduced for DEG rather than for DEP.

Complementary to this descriptive analysis, the data integration method MFA was used to highlight the most important contributors to RFI difference. Details for this mathematical method that analyses the relationships between tissue-wise probe sets can be read elsewhere [55]. Its ability to summarize relevant biological messages across separate transcriptomic datasets was recently illustrated in pig nutritional studies [53]. The FactoMineR package in R software was used. Four principal component analyses (PCA) were first calculated from the DEP dataset (one for each tissue), and each variable identified by the probe name was weighed by the first eigenvalue in each PCA. The next analysis consisted in performing a meta-PCA by using these weighed variables as new entries, thus ensuring that each tissue influence was equally distributed to analyze the whole transcriptomic variability across tissues. Thereby, the three first axes of these four separate PCA (Dim_*i*_muscle, Dim_*i* Liver, Dim_*i*_PRAT and Dim_*i*_SCAT with *i* = 1 to 3) were projected as supplementary variables in the principal MFA plane (Dim1; Dim2). Calculating the correlations between each DEP and the first two MFA dimensions allows deciphering the DEP which were mainly responsible for variability in overall molecular responses. In the present study, the retained correlation threshold was r > |0.70| (*P* < 0.001).

### Functional pathways analysis

The gene ontology terms for biological processes (GOBP) were automatically searched within lists of DEG, using the Database for Annotation, Visualization and Integrated Discovery (DAVID) bioinformatics resource database (http://david.abcc.ncifcrf.gov/ [56]). This analysis can be considered as a statistical method that exploits a priori knowledge to provide a biological meaning to the identified genes and reduce the multiple-testing burden [21]. The results were downloaded using the “Functional annotation clustering” option of the DAVID tool. The enrichment (E) score (which is measured by the geometric mean of the EASE score of all enriched annotations terms) and the modified Fisher exact *P*-value were obtained. In this study, E ≥ 1 and *P*-value ≤ 0.05 were retained to list the groups of genes. Plausible regulatory networks that included expression changes were then visualized using QIAGEN’s Ingenuity® Pathway Analysis (IPA®, QIAGEN Redwood City, CA, www.qiagen.com/ingenuity), with the main goals to generate functional hypotheses and identify central molecules in small sub-networks.

### Quantitative real-time PCR (qPCR)

Technical validation of microarrays data was performed by examining expression levels of target genes by qPCR. First-strand cDNA synthesis was performed with 1 μg of total RNA used for microarray analysis (*n =* 48 pigs) in the four tissues, by using High Capacity RNA to cDNA Kit (Applied Biosystems, Foster City, USA). Primers were designed from porcine sequences available in Ensembl or NCBI databases using Primer Express® v3.0 software (Applied Biosystems). Detailed information for primer sequences (forward and reverse) is provided in Additional file 2. Amplification reactions and disassociation curves were carried out on a StepOnePlus^TM^ real-time PCR system (Applied Biosystems). As stated by the GenNorm algorithm among three other tested reference genes, *TOP2B* gene was identified as the most stable housekeeping gene and was used for normalization. Using R software, the ANOVA model used for microarray analyses was then applied to determine significant effect of line on gene expression levels.

## Additional files

Additional file 1: Number of differentially expressed probes and unique genes in tissues between pigs from the low RFI or high RFI lines. (DOCX 24 kb)

Additional file 2: Lists of genes differentially expressed between pigs from the low or high RFI lines in muscle, liver and two adipose tissues. (XLSX 366 kb)

Additional file 3: Comparison between microarrays and qPCR for target genes. (DOCX 26 kb)

Additional file 4: Differentially expressed genes shared by muscle, liver and two adipose tissues between pigs from the low or high RFI lines. (XLSX 31 kb)

Additional file 5: Networks of genes involved in immune response (DOCX 658 kb)

Additional file 6: Network of genes encompassing intracellular signaling pathways (DOCX 346 kb)

Additional file 7: Top molecular contributors to RFI difference across tissues as indicated a multiple factor analysis (MFA). (DOCX 28 kb)

Additional file 8: Genes participating to relevant GO biological processes specifically in longissimus muscle. (DOCX 28 kb)

Additional file 9: Genes participating to relevant GO biological processes specifically in liver. (DOCX 22 kb)

Additional file 10: Genes participating to relevant GO biological processes specifically in adipose tissues. (DOCX 22 kb)

## Abbreviations

ADG: Average daily gain
BFT: Backfat thickness
BW: Body weight
DAVID: Database for annotation, visualization and integrated discovery
DEG: Differentially expressed gene
DEP: Differentially expressed probe
DFI: Daily feed intake
Dim: Dimension
FCR: Feed conversion ratio
G:F: Gain-to-feed ratio
GOBP: Gene ontology biological process
MFA: Multiple factor analysis
PCA: Principal component analysis
PRAT: Perirenal adipose tissue
qPCR: quantitative real-time polymerase chain reaction
RFI: Residual feed intake
SCAT: Subcutaneous adipose tissue.
