# Supplementary material for: A transcriptome multi-tissue analysis identifies biological pathways and genes associated with variations in feed efficiency of growing pigs
Source: BMC Genomics. 2017 Mar 21;18:244. doi: 10.1186/s12864-017-3639-0 (PMC5361837; doi:10.1186/s12864-017-3639-0)
Supplement: Supplementary file 1 — Number of differentially expressed probes and unique genes in tissues between pigs from the low RFI or high RFI lines. (DOCX 24 kb) [file 12864_2017_3639_MOESM1_ESM.docx]

**Additional file 1 Number of differentially expressed probes and unique genes in tissues between pigs from the low RFI or high RFI lines**

|  | Low/high RFI lines | |
| --- | --- | --- |
|  | Over-expressed | Under-expressed |
| **Muscle** |  |  |
| *P* < 0.01 |  |  |
| With FC^1^ ≥ \|1.1\| | 3241 (1552) | 2167 (942) |
| With FC ≥ \|1.5\| | 95 (47) | 288 (70) |
| With FC ≥ \|2\| | 30 (11) | 48 (9) |
| *P*-BH <5% | 3148 (1512) | 2083 (905) |
| *P*-BH <10% | 3241 (1552) | 2167 (942) |
| **Liver** |  |  |
| *P* < 0.01 |  |  |
| With FC ≥ \|1.1\| | 1224 (579) | 1164 (522) |
| With FC ≥ \|1.5\| | 134 (56) | 162 (56) |
| With FC ≥ \|2\| | 34 (14) | 22 (8) |
| *P*-BH <5% | 693 (335) | 691 (297) |
| *P*-BH <10% | 999 (469) | 973 (439) |
| **PRAT** |  |  |
| *P* < 0.01 |  |  |
| With FC ≥ \|1.1\| | 983 (467) | 1027 (446) |
| With FC ≥ \|1.5\| | 119 (49) | 115 (33) |
| With FC ≥ \|2\| | 35 (15) | 14 (4) |
| *P*-BH <5% | 504 (203) | 482 (232) |
| *P*-BH <10% | 729 (315) | 724 (351) |
| **SCAT** |  |  |
| *P* < 0.01 |  |  |
| With FC ≥ \|1.1\| | 780 (370) | 616 (276) |
| With FC ≥ \|1.5\| | 83 (32) | 64 (19) |
| With FC ≥ \|2\| | 27 (13) | 7 (2) |
| *P*-BH <5% | 180 (53) | 110 (85) |
| *P*-BH <10% | 325 (103) | 217 (158) |

^1^FC: fold-change between mean values calculated in pigs of the low vs. high RFI lines. Values are inversed and preceded by a minus sign for FC < 1 (e.g., FC = 0.5 was indicated as FC = -2). Probes were declared as differentially-expressed between pigs of the low or high RFI lines when FC > |1.1| and *P* < 0.01. Number of probes elicited after Benjamini-Hochberg correction for multiple tests (*P*-BH) was also indicated. Corresponding numbers in differentially-expressed unique genes are indicated into brackets. When several probes were differentially expressed for a unique gene, the gene was retained if at least one probe met the cut-off.
