# Supplementary material for: A transcriptome multi-tissue analysis identifies biological pathways and genes associated with variations in feed efficiency of growing pigs
Source: BMC Genomics. 2017 Mar 21;18:244. doi: 10.1186/s12864-017-3639-0 (PMC5361837; doi:10.1186/s12864-017-3639-0)
Supplement: Supplementary file 3 — Comparison between microarrays and qPCR for target genes. (DOCX 26 kb) [file 12864_2017_3639_MOESM3_ESM.docx]

**Additional file 3 Comparison between microarrays and qPCR for target genes**

|  | FC | FC | forward (f) and reverse (r) primers in qPCR |
| --- | --- | --- | --- |
| Gene ID | Microarray | qPCR |  |
|  | **Muscle** |  |  |
| YME1L1 | 0.88 | 0.88 | f:CAATGCCTATCAACAAAGCTACGA; r:CAGGCAACAGGGACACATGT |
| SOD2 | 0.87 | 0.91 | f:GCGCTGAAAAAGGGTGATGT; r:ACCGTTAGGGCTCAGATTTGTC |
| GPX3 | 0.81 | 0.71 | f:GCTTCCCCTGCAACCAATT; r:GGACATACCTGAGAGTGGACAGAA |
| OAZ3 | 0.81 | 0.23 | f:GGCAACCGGGAAAGTTTAACT; r:CGTAGCAAGTCACCTCTGTCATTC |
| MYC | 0.75 | 0.72 | f:CGGAGAGGCTATTCTGCCTATTT; r:AGCCTGCGGGTTTTCCA |
| MYOD1 | 0.83 | 0.83 | f:GCCGCTTGAGCAAAGTCAA; r:CGCTGATTCGGGTTGCTAGA |
| GALNT | 0.87 | 0.90 | f:TGCTCAAATGCCACCACCTA; r:GGCTTTGTCCAGGCACTGAT |
| ME1 | 0.76 | 0.86 | f:TGGTGACTGATGGAGAACGTATTC; r:CAGGATGACAGGCAGACATTCTT |
| PPARD | 1.20 | 0.97 | f:CGCATGAAGCTGGAGTACGA; r:AGCGAATGGCGTTGTGAGA |
| B4GALT5 | 0.84 | 0.92 | f:TGCTTACGCCAAGAGGAACA; r:TTTCGCTGTGGTTCAAGTCAA |
| NFYB | 1.01 | 0.97 | f:GGGAATTGGTGGAGCAGTCA; r:CAACTGGTTCGTAAATGCCTCTT |
| SMARCA1 | 0.86 | 0.86 | f:GCAAGTGCTGATGTCGTTATACTATATG; r:CCCGATCCATTGCTTGTAGATC |
|  | **Liver** |  |  |
| YME1L1 | 0.84 | 0.90 | f:CAATGCCTATCAACAAAGCTACGA ;r:CAGGCAACAGGGACACATGT |
| NFKBIA | 0.81 | 0.78 | f:CCAACTACAATGGCCACACATG; r:AAAGACACCAACAGCTCCACAA |
| NFYB | 1.19 | 1.29 | f:GGGAATTGGTGGAGCAGTCA; r:CAACTGGTTCGTAAATGCCTCTT |
| IRF1 | 0.80 | 0.82 | f:AGCACCAGCGACCTGTACAACT; r:TCCTCATCTGTTGCAGCTTCAG |
| ABCF1 | 1.37 | 1.07 | f:GCCTGCATGGTGTGACATTT; r:ATGCCGAAGTCCAGATTCTTAAAG |
| FAAH | 1.29 | 1.35 | f:CACACGCTGATTCCCTTCCT; r:TGTAGCAACCTCTTCCCACCAT |
| MAN2C1 | 0.87 | 0.93 | f:AATGCAAGAGACGGATGGTTCT; r:GTGCCCTCAGCAATGACCTC |
| NLRC5 | 0.68 | 0.74 | f:CAAGTCATTCGCCTGTGGAA; r:GCTGATGGTCGAAGAAAGCAA |
| PPARA | 1.10 | 1.10 | f:AAGGTTGCAAGGGCTTCTTTC; r:CTTACAGCTCCGATCACATTTGTC |
| PPP1R1A | 0.77 | 0.84 | f:CCACGGCAACAGAAGAAGGT; r:CCATCATCTGGAGCTCTTTCATT |
| RARA | 0.97 | 0.97 | f:CAAGACAAATCCTCAGGCTACC; r:CACCATGTTCTTCTGGATGC |
| CEBPB | 0.79 | 0.84 | f:AACCTGGAGACGCAGCATAAG; r:TCTTCTGGAGCCGCTCGTT |
|  | **PRAT** |  |  |
| YME1L1 | 0.86 | 0.89 | f:CAATGCCTATCAACAAAGCTACGA ;r:CAGGCAACAGGGACACATGT |
| SOD2 | 1.53 | 1.20 | f:GCGCTGAAAAAGGGTGATGT; r:ACCGTTAGGGCTCAGATTTGTC |
| NFE2L2 | 1.07 | 1.07 | f:GCACAACACATCCCGTCAGA; r:GAATGTGGGCTACCTGGGAAT |
| GALNT1 | 0.82 | 0.96 | f:TGCTCAAATGCCACCACCTA; r:GGCTTTGTCCAGGCACTGAT |
| FAAH | 1.77 | 1.96 | f:CACACGCTGATTCCCTTCCT; r:TGTAGCAACCTCTTCCCACCAT |
| SREBF2 | 0.88 | 0.92 | f:GGAGGAGGAGAGCTGTGAATTC; r:CCCCACAGAGTCCACAAAAGA |
| RARA | 0.95 | 0.95 | f:CAAGACAAATCCTCAGGCTACC; r:CACCATGTTCTTCTGGATGC |
| PPARG | 1.02 | 1.22 | f:ATTCCCGAGAGCTGATCCAA; r:TGGAACCCCGAGGCTTTAT |
| MTSS1 | 1.49 | 1.06 | f:CGGCATGTTCCCGTCATC; r:CACCAGAGGCTGATCGTAAGGT |
| RUNX1T1 | 0.82 | 0.95 | f:GAACTCCAGACAGAACCAAAGAAAAT; r:CGCTTGCTTGGATGTTCTGA |
| STAM2 | 0.89 | 1.01 | f:GTTCCAGTGCAGACATATCCAGTT; r:TCTGGGCAACGGTGACTTG |
|  | **SCAT** |  |  |
| YME1L1 | 0.90 | 0.94 | f:CAATGCCTATCAACAAAGCTACGA ;r:CAGGCAACAGGGACACATGT |
| SOD2 | 1.25 | 1.27 | f:GCGCTGAAAAAGGGTGATGT; r:ACCGTTAGGGCTCAGATTTGTC |
| NFE2L2 | 1.06 | 1.02 | f:GCACAACACATCCCGTCAGA; r:GAATGTGGGCTACCTGGGAAT |
| FAAH | 1.74 | 1.84 | f:CACACGCTGATTCCCTTCCT; r:TGTAGCAACCTCTTCCCACCAT |
| NRF1 | 1.08 | 1.08 | f:CGGCCTCATGTGTTTGAGTCT; r:TGGCTCGAAGTTTCCTAAGCA |
| STAM2 | 0.87 | 1.02 | f:GTTCCAGTGCAGACATATCCAGTT; r:TCTGGGCAACGGTGACTTG |
| A subset of genes declared as differentially expressed or as not different between RFI groups by statistical analysis of microarray data were analyzed by qPCR.  Correlation between microarray and qPCR data | | | |
| FC: fold-change calculated between low to high RFI pigs | | | |
