# Supplementary material for: A transcriptome multi-tissue analysis identifies biological pathways and genes associated with variations in feed efficiency of growing pigs
Source: BMC Genomics. 2017 Mar 21;18:244. doi: 10.1186/s12864-017-3639-0 (PMC5361837; doi:10.1186/s12864-017-3639-0)
Supplement: Supplementary file 5 — Networks of genes involved in immune response (DOCX 658 kb) [file 12864_2017_3639_MOESM5_ESM.docx]

**Additional file 5 Networks of genes involved in immune response^1^**

**A-
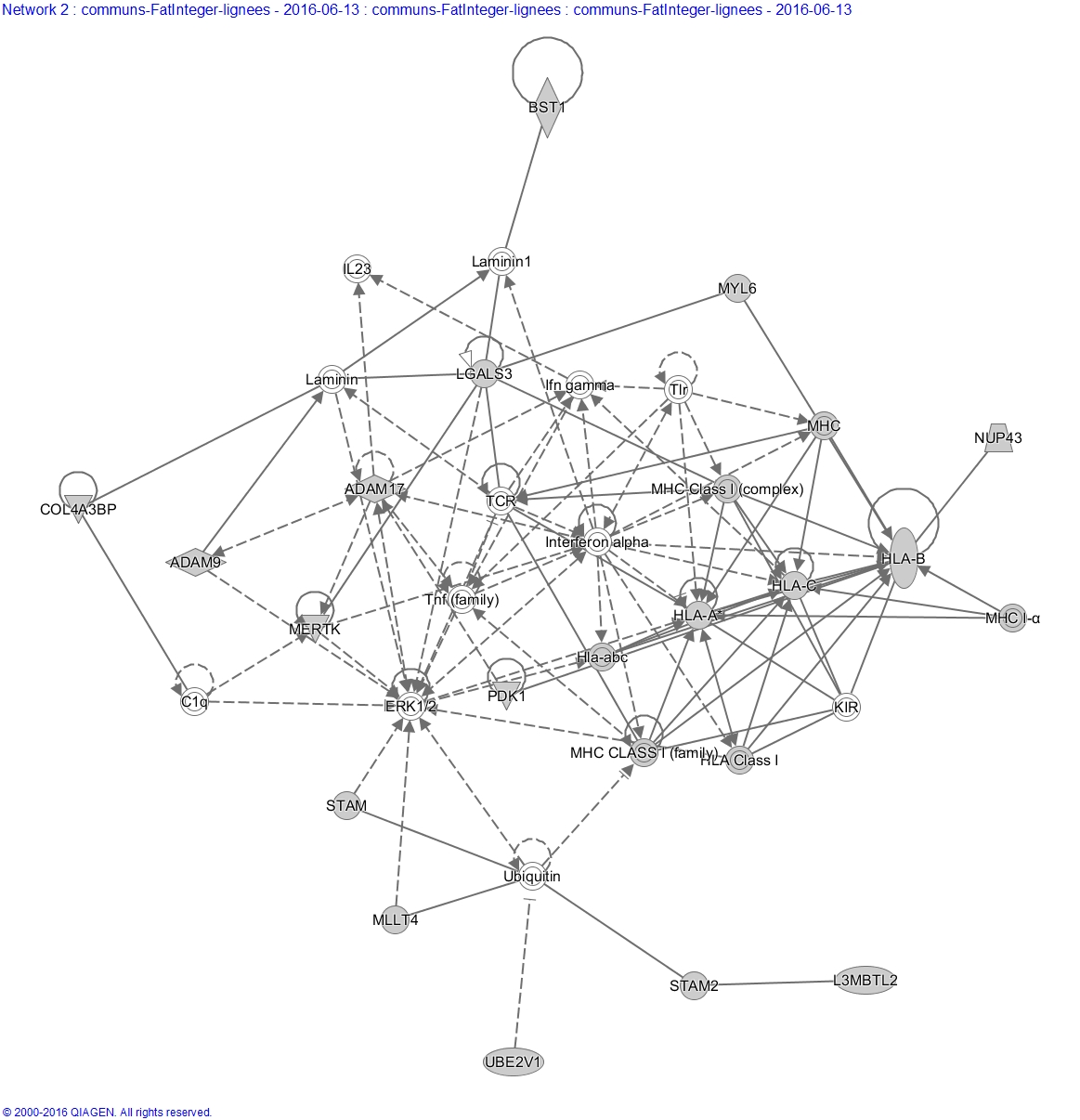
**

**B-
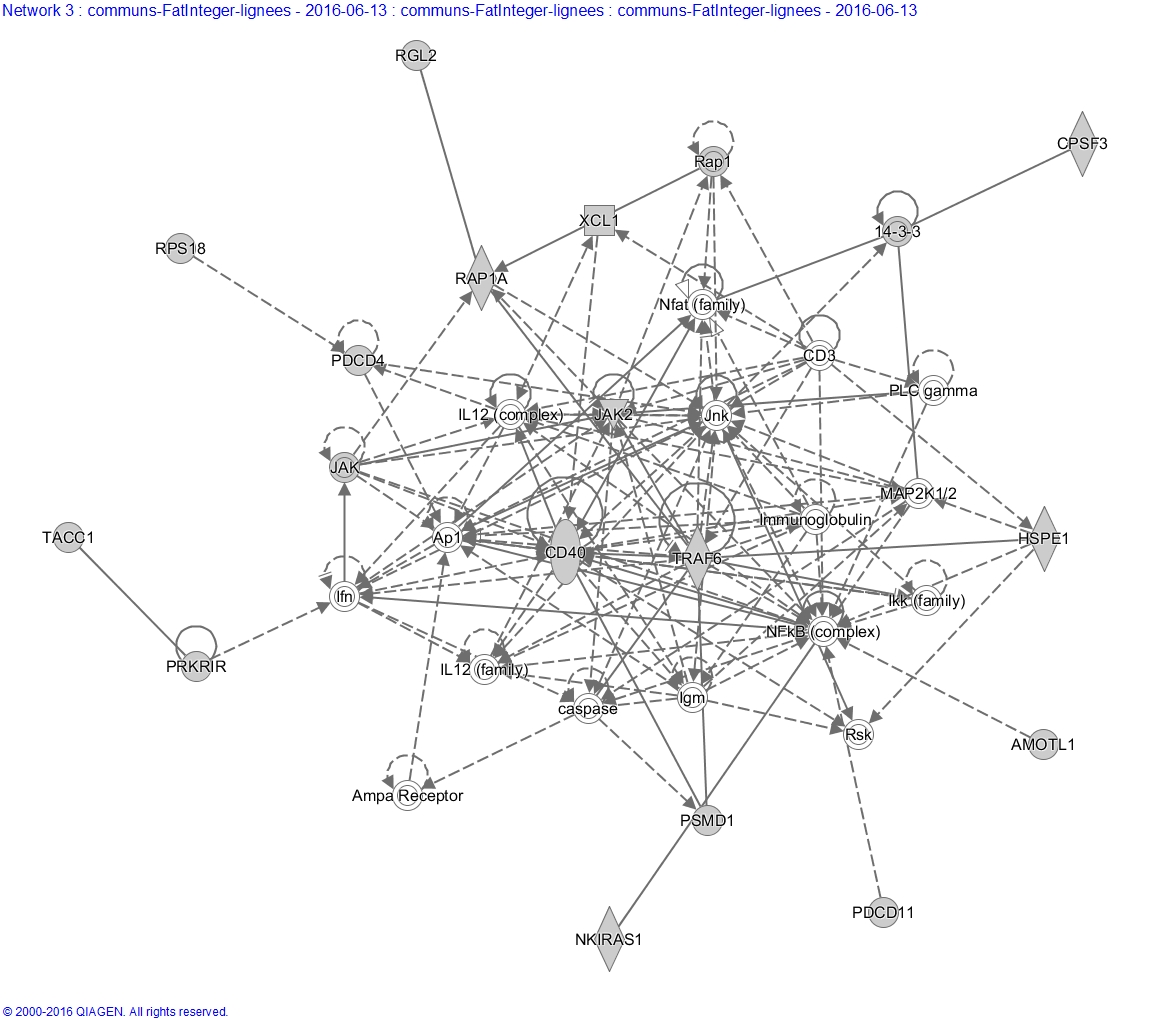
**

Members of the major histocompatibility complex family (*HLA-A/B/C*) were associated with the phosphoinositide protein kinase *(PDK1*) participating in lymphocyte homeostasis and function, and this notably pointed to interferon signaling (A). Involvement of *CD40* and *TRAF6* (pro-inflammatory roles) in C-jun N-terminal kinase (JNK) and nuclear factor kappa-light-chain-enhancer of activated B cells (NFκB) signaling pathways were suggested (B).
