# Supplementary material for: A transcriptome multi-tissue analysis identifies biological pathways and genes associated with variations in feed efficiency of growing pigs
Source: BMC Genomics. 2017 Mar 21;18:244. doi: 10.1186/s12864-017-3639-0 (PMC5361837; doi:10.1186/s12864-017-3639-0)
Supplement: Supplementary file 6 — Network of genes encompassing intracellular signaling pathways (DOCX 346 kb) [file 12864_2017_3639_MOESM6_ESM.docx]

**Additional file 6 Network encompassing intracellular signaling pathways^1^**

**
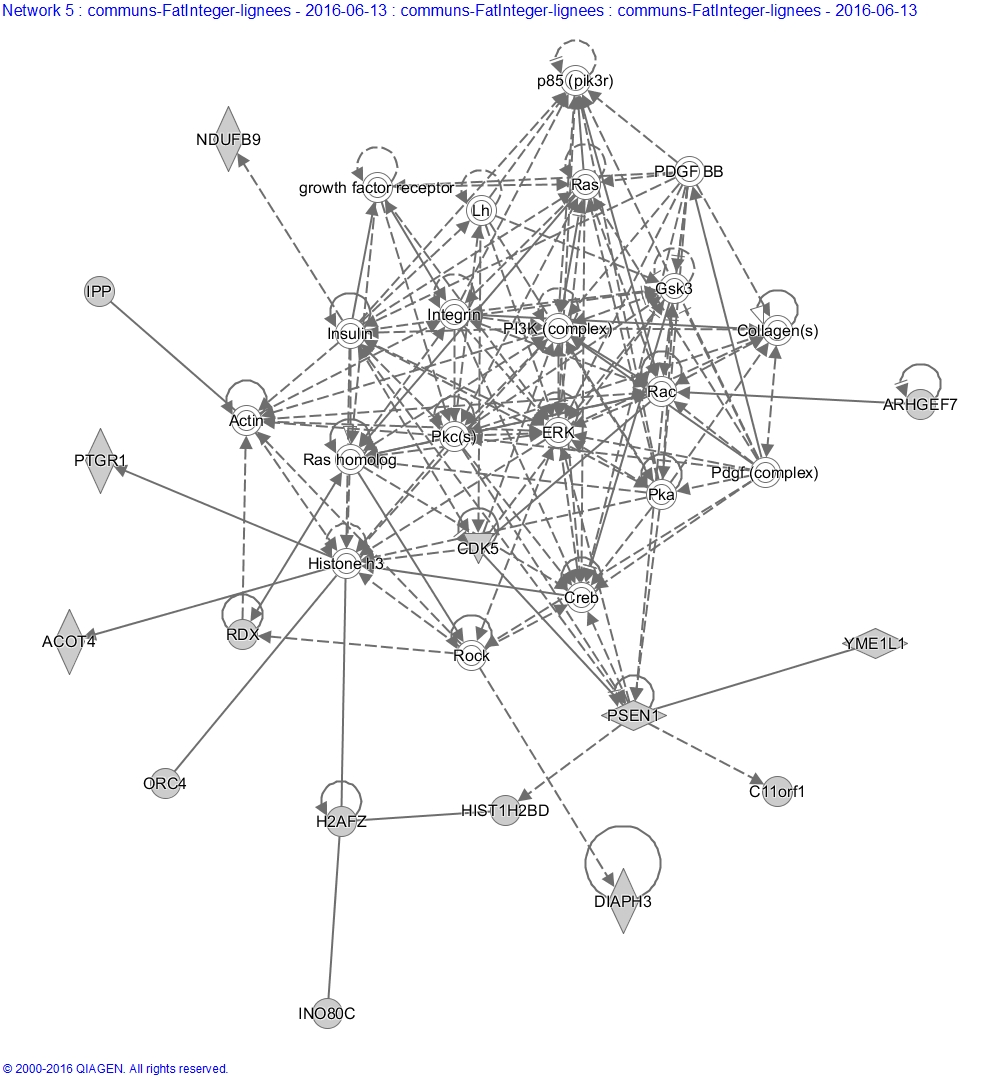
**

**^1^**Phosphoinositide 3-kinase (PI3K) and ERK signaling pathways were put on evidence with possible connections with *CREB1*, a gene known to induce transcription of genes of fatty acid metabolism*.* In the same network, *YME1L1*, a gene involved in mitochondrial protein metabolism, was associated with *PSEN1* a gene that may regulate protease enzymes.
