# Supplementary material for: A transcriptome multi-tissue analysis identifies biological pathways and genes associated with variations in feed efficiency of growing pigs
Source: BMC Genomics. 2017 Mar 21;18:244. doi: 10.1186/s12864-017-3639-0 (PMC5361837; doi:10.1186/s12864-017-3639-0)
Supplement: Supplementary file 7 — Top molecular contributors to RFI difference across tissues as indicated a multiple factor analysis (MFA). (DOCX 28 kb) [file 12864_2017_3639_MOESM7_ESM.docx]

**Additional file 7** Top molecular contributors in the first dimension of the multiple factor analysis (MFA)

Only the molecular probes showing a correlation coefficient > 0.70 with the first MFA dimension and commonly found in muscle, liver and two adipose tissues were indicated

| Probe name | Gene symbol | r | *P*-value |
| --- | --- | --- | --- |
| **Associated with low RFI** | | | |
| A_72_P304024 | PSEN1 | 0.845 | 4.44E-14 |
| O12773 | PCIF1 | 0.773 | 1.20E-10 |
| A_72_P722393 | RPL6 | 0.771 | 1.42E-10 |
| A_72_P585246 | PCIF1 | 0.769 | 1.80E-10 |
| A_72_P109781 | IPP | 0.767 | 2.07E-10 |
| A_72_P614716 | RPL14 | 0.765 | 2.41E-10 |
| A_72_P719128 | RPL6 | 0.762 | 3.10E-10 |
| A_72_P655183 | RPL14 | 0.761 | 3.56E-10 |
| A_72_P761955 | RPL6 | 0.758 | 4.55E-10 |
| A_72_P680165 | PHYH | 0.756 | 5.39E-10 |
| O318 | PLA2G12A | 0.755 | 5.86E-10 |
| A_72_P337268 | HEATR4 | 0.754 | 6.07E-10 |
| A_72_P585246 | PCIF1 | 0.748 | 1.01E-09 |
| A_72_P016041 | unknown | 0.746 | 1.18E-09 |
| A_72_P011181 | ZNF174 | 0.745 | 1.21E-09 |
| A_72_P440081 | PHYH | 0.744 | 1.33E-09 |
| O10419 | USP33 | 0.741 | 1.74E-09 |
| O9386 | MRPL9 | 0.740 | 1.92E-09 |
| O12773 | PCIF1 | 0.738 | 2.24E-09 |
| A_72_P011181 | ZNF174 | 0.736 | 2.48E-09 |
| A_72_P204642 | RPL14 | 0.736 | 2.53E-09 |
| A_72_P683539 | RPL14 | 0.735 | 2.61E-09 |
| A_72_P734143 | RPL6 | 0.735 | 2.80E-09 |
| O1933 | PIGL | 0.734 | 2.90E-09 |
| A_72_P683539 | RPL14 | 0.733 | 3.07E-09 |
| A_72_P563904 | RPL14 | 0.731 | 3.68E-09 |
| A_72_P441723 | RPL14 | 0.729 | 4.16E-09 |
| A_72_P647143 | PHYH | 0.728 | 4.40E-09 |
| A_72_P016041 | unknown | 0.727 | 4.80E-09 |
| A_72_P563904 | RPL14 | 0.727 | 4.82E-09 |
| A_72_P152921 | MTHFS | 0.725 | 5.75E-09 |
| A_72_P599398 | PHYH | 0.724 | 5.99E-09 |
| O2068 | C19orf42 | 0.724 | 6.10E-09 |
| O3529 | HEATR4 | 0.723 | 6.55E-09 |
| A_72_P337268 | HEATR4 | 0.722 | 7.04E-09 |
| A_72_P756548 | RPL6 | 0.722 | 7.07E-09 |
| A_72_P305509 | TMEM181 | 0.721 | 7.54E-09 |
| A_72_P305509 | TMEM181 | 0.720 | 8.14E-09 |
| A_72_P729538 | RPL6 | 0.719 | 8.73E-09 |
| A_72_P498903 | MTHFS | 0.717 | 1.00E-08 |
| A_72_P199102 | PHYH | 0.717 | 1.02E-08 |
| A_72_P376878 | PGPEP1 | 0.716 | 1.08E-08 |
| A_72_P325613 | C8orf37 | 0.711 | 1.50E-08 |
| A_72_P655183 | RPL14 | 0.710 | 1.55E-08 |
| A_72_P278789 | C8orf37 | 0.710 | 1.64E-08 |
| A_72_P120586 | C6orf106 | 0.709 | 1.70E-08 |
| A_72_P225367 | TFB1M | 0.705 | 2.15E-08 |
| **Associated with high RFI** | | | |
| A_72_P040016 | unknown | -0.858 | 6.94E-15 |
| A_72_P294444 | CD40 | -0.791 | 2.22E-11 |
| A_72_P115846 | NTN1 | -0.782 | 5.43E-11 |
| A_72_P087836 | unknown | -0.782 | 5.47E-11 |
| A_72_P124966 | CD40 | -0.781 | 5.82E-11 |
| A_72_P088376 | CD40 | -0.768 | 1.86E-10 |
| A_72_P515641 | POLR3H | -0.766 | 2.26E-10 |
| O10452 | LZTFL1 | -0.764 | 2.70E-10 |
| A_72_P515641 | POLR3H | -0.762 | 3.04E-10 |
| A_72_P231852 | unknown | -0.759 | 3.95E-10 |
| A_72_P048381 | unknown | -0.753 | 6.72E-10 |
| A_72_P205717 | CYBASC3 | -0.749 | 9.53E-10 |
| A_72_P368058 | H2-Q4 | -0.748 | 9.76E-10 |
| A_72_P043191 | PIKFYVE | -0.744 | 1.36E-09 |
| O5247 | CTSC | -0.743 | 1.46E-09 |
| A_72_P372208 | DST | -0.740 | 1.92E-09 |
| O2995 | UQCRB | -0.739 | 2.01E-09 |
| A_72_P414198 | unknown | -0.737 | 2.25E-09 |
| A_72_P515641 | POLR3H | -0.736 | 2.53E-09 |
| A_72_P040016 | unknown | -0.735 | 2.76E-09 |
| A_72_P414198 | unknown | -0.735 | 2.80E-09 |
| A_72_P442498 | SMYD3 | -0.734 | 2.96E-09 |
| A_72_P040016 | unknown | -0.733 | 3.06E-09 |
| gi\|115554271\|dbj\|AK240066.1\| | CTSC | -0.732 | 3.46E-09 |
| A_72_P404073 | LRCH1 | -0.730 | 3.88E-09 |
| A_72_P227892 | unknown | -0.730 | 3.92E-09 |
| A_72_P427959 | MUTYH | -0.727 | 4.76E-09 |
| O9703 | TACC1 | -0.727 | 4.99E-09 |
| gi\|115548138\|dbj\|AK238122.1\| | DST | -0.724 | 6.04E-09 |
| A_72_P599398 | PHYH | -0.723 | 6.38E-09 |
| A_72_P470830 | ORC4 | -0.720 | 7.94E-09 |
| O10452 | LZTFL1 | -0.716 | 1.04E-08 |
| A_72_P285984 | unknown | -0.714 | 1.23E-08 |
| CUST_334_PI427286967 | TACC1 | -0.714 | 1.23E-08 |
| O10918 | SLCO2B1 | -0.713 | 1.26E-08 |
| A_72_P199102 | PHYH | -0.712 | 1.41E-08 |
| A_72_P048381 | unknown | -0.711 | 1.47E-08 |
| A_72_P043191 | PIKFYVE | -0.709 | 1.73E-08 |
| A_72_P517623 | HLA-A | -0.709 | 1.76E-08 |
| A_72_P087836 | unknown | -0.707 | 1.96E-08 |
| A_72_P394458 | MLX | -0.707 | 2.01E-08 |
| O9607 | HLA-B | -0.705 | 2.23E-08 |
| A_72_P337183 | N4BP2 | -0.704 | 2.41E-08 |
| gi\|115555467\|dbj\|AK234080.1\| | NMI | -0.703 | 2.62E-08 |
| A_72_P647143 | PHYH | -0.700 | 3.01E-08 |
|  | | | |
