# Supplementary material for: A transcriptome multi-tissue analysis identifies biological pathways and genes associated with variations in feed efficiency of growing pigs
Source: BMC Genomics. 2017 Mar 21;18:244. doi: 10.1186/s12864-017-3639-0 (PMC5361837; doi:10.1186/s12864-017-3639-0)
Supplement: Supplementary file 8 — Genes participating to relevant GO biological processes specifically in longissimus muscle. (DOCX 28 kb) [file 12864_2017_3639_MOESM8_ESM.docx]

**Additional file 8** *Genes participating to relevant GO biological processes in longissimus muscle as affected by selection for RFI*

| Biological processes^1^ | Genes^2^ |
| --- | --- |
| **Muscle:** Overexpressed in low RFI pigs compared with high RFI pigs | |
| GO:0006412~translation | RPLP0, RPLP1, RPL4, RPL5, RPL6, RPL7, RPL8, RPL10, RPL10A, RPL11, RPL12, RPL13AP3, RPL13AP5, RPL14, RPL15, RPL18, RPL21, RPL21P28, RPL22, RPL23, RPL23A, RPL24, RPL26L1, RPL27, RPL28, RPL29, RPL31, RPL32, RPL34, RPL35, RPL36, RPL36A, RPL36AL, RPL37A, RPL41, MRPL21, MRPL52, RPS3, RPS3A, RPS4X, RPS6, RPS7, RPS8, RPS11, RPS12, RPS13, RPS14, RPS15, RPS15A, RPS16, RPS17, RPS18, RPS19, RPS21, RPS23, RPS20, RPS24, RPS26, RPS29, RPSA, RPSAP9, RPSAP58, MRPS11, MRPS18B, MRPS18C, EIF1B, EIF2A, EIF2S2, EIF3E, EIF3F, EIF3H, EIF3I, EIF3K, EIF4B, EIF4A2, EEF1A2, EEFSEC, EIF2AK4, EEF1G, EEF1D, EEF2,UBA52, PABPC4, MTIF2, KARS, PAIP1, DALRD3, EPRS, MRRF, ABCF1, COPS5, DPH1, NARS2, TPR, MTRF1, LARS, AIMP1, HBS1L, FAU, CARS |
| GO:0022613~ribonucleoprotein complex biogenesis | RPLP0, RPL7, RPL5, RPL14, RPL11, RPL24, RPS24, RPS6, RPS7, RPS19, RPS16, RPS17, RPS14, RPS15, RBM5, RRS1, EIF2A, MINA, SBDS, NSA2, KRR1, EXOSC9, TSR1, SNUPN, BYSL, DDX1, MPHOSPH10, MBNL1, TSC1, FBLL1, ZNHIT6, SNRPG, SNRPD3 |
| GO:0003012~muscle system (contraction) process | MYOT, MYOM2, MYBPC2, TTN, TNNC2, TNNI2, TNNT3, MYH4, MYH8, RYR1, RYR2, RYR3, PGAM2, RPS6KB1, KCNE1, MAP2K6, , MAP2K3, PABPN1, SMAD5, ARHGEF11, TRDN, CACNA1G, TMOD4, KBTBD10, ALDOA |
| GO:0006091~generation of precursor metabolites and energy  (GO:0006006~glucose metabolic process) | INS-IGF2, IRS2, HK3, PPP1R2P3, PPP1R3A, PPP1CB, PRKAG3, PYGM, GSK3B, AGL, ALDOA, GAPDH, GAPDHS, PGM1, PGAM1, PGAM2, ENO3, IDH3G, PGM2L1, PHKA1, PDK3, DLAT, COX7C, COX17, ND4L, ND4, ND3, NDUFA5, NDUFB11, NDUFA2, NDUFS8, NDUFA12, NDUFV3, ATP5G2, MCHR1, PPARD, UQCRC1, UQCRB, FECHIDH3B, RBP4, TNF, AIMP1, MAPK14 |
| GO:0032268~regulation of cellular protein metabolic process  (GO:0006417~regulation of translation) | INS-IGF2, INSR, IGF2BP2, GHR, RPS4X, EIF2A, EIF1B, EIF3H, EIF3E, EIF3K, EIF4B, EIF4A2, EIF2AK4, EEFSEC, PSMD1, PSMD13, PSMB6, PSME1, PSEN1, YWHAE, RNF139, UBA52, TNF, PDGFA, IMPACT, IDE, PAX5, YBX2, PRKAR2A, ATG7, PUM1, DNAJC1, SAMD4A, SOCS1, PKN1, MAP4K5, MTRF1, NCK1, PDGFRB, ANAPC11, MTIF2, PIN1, ITGAV, BMP4, BMPR1A, MDFI, PAIP1, DDX1, TSC1, JAK2, TNK2, VPS28 |
| GO:0030163~protein catabolic process  (GO:0006511~ubiquitin-dependent catabolic process) | PSMB6, PSMD1, PSMD13, PSME1, PSEN1, UBA52, UBE2Q2, UBE2Q1, UBE2M, UBE2J2, UBE2O, USP7, USP8, USP13, USP14, USP15, USP33, USP47, USP48, USE1, SENP5, SENP7, CUL3, CUL5, CUL9, DERL1, DERL3, RNF7, RNF14, RNF139, NEURL2, FBXL4, FBXO7, FBXO9, FBXO31, FBXO45, FBXW4, TRAF6, WWP2, ATG7, ITCH, AUP1, PAN2, DTL, SOCS1, TOPORS, HERC2, RFWD2, DCUN1D2, ATG4D, ASB1, SIAH2, KBTBD10, CUEDC2, ANAPC11, C12ORF51, ASB15, ACE, TPRKB, SHPRH, LRRC41, PARK2, MARCH5, TULP4, KCMF1, PPP2R5C, IDE |
| GO:0046907~intracellular transport  (GO:0008104~protein localization) | YWHAE, YWHAG, PSEN1, RAB3GAP2, CLTB, USE1, VPS37A, RAB1B, MXI1, TGFB2, KIF13A, TRIM3, LONP2, PEX1, ATG7, RPL11, STAM, VPS13A, VPS16, NUP35, TPR, TOMM34, ALS2CL, CUTA, SEC23A, ACTN4, SNUPN, STXBP2, VTI1B, STXBP3, TOPORS, HERC2, IPO8, VTI1A, PRKCB, RPAIN, ATG4D, IPO5, SORT1, TOMM22, RAB12, PDCD6IP, KPNA4, KPNA3, SEC23B, ARFGAP1, SNX9, RAB3A, TNF, DERL1, EXOC5, EXOC7, EXOC8, STAM2, NFKBIB, SNX16, AKAP12, RDX, SEC62, CEP57, BCAP29, RAB11A, TMED10, SUPT7L, TRAM1, TNPO1, TERF2, TNPO3, HSPA9, AP2M1, MDFI, NUP155, NFKBIL1, GABARAP, COG5, RPL23, TOMM70A, GSK3B, JAK2, GOSR1, VPS28, SSR4, SSR2, NR5A1 |
| GO:0005977~glycogen metabolic process | PRKAG3, PYGM, GSK3B, PPP1R3A, PPP1R2P3, PPP1CB, PHKA1, AGL |
| GO:0046324~regulation of glucose import | INSR, IRS2, INS-IGF2, GSK3A, SORBS1, APPL1, TNF, LTA, LTB, NFKBIL1 |
| **Muscle:** Under-expressed in low RFI pigs compared with high RFI pigs | |
| GO:0006955~immune response  (GO:0006952~defense response) | HLA-DRB3, IL18, TLR1, TLR2, TLR4, C1QB, C1QC, CYBA, CYBB, TNFRSF1B, TNFAIP6, TGFB1, C2, C4BPA, CFI, CD1A, CD14, CD48, CD83, CD86, CD97, CD163, CD302, BCL10, B2M, CCL2, CTSS, LY86, LY96, LYN, IFNA5, GNL1, NCF2, NCF4, NFIL3, SERPING1, FCGR3B, EBI3, GEM, IGSF6, CCR5, MADCAM1, TREM2, VSIG4, GPR183, C3CLU, GPSM3, DEFB4A, ENPP1, IGF1R, SLC11A1, XBP1, ICOS, BCL2, FCGR1A, TAP1, FCER1G, THBS1, BLNK, SWAP70, MSH2, VAV1, CCL13, FCGR2B, AIF1, HMOX1, MX2, IRAK2, PDPN, LYZ, MYD88, CST3, NLRP1, F3, CX3CR1, RIPK2, NFATC3, FN1, TYROBP, LIPA, RNASE6, ANXA1 |
| GO:0002250~adaptive immune response | IL18, , TLR4, C1QB, C1QC, C2, C3, C4BPA, CFI, BCL10, SWAP70, MSH2, CLUSERPING1, SLC11A1, MYD88, FCER1G, GNL1, EBI3 |
| GO:0001817~regulation of cytokine production | IL18, TLR1, TLR2, TLR4, TGFB1, CD14, CD83, CD86, IFNAR1, HMOX1, CASP1, BCL10, SLC11A1, PRKCQ, MYD88, RIPK2, FCER1G, THBS1, VSIG4, ATP6AP2, EBI3, SYK |
| GO:0010033~response to organic substance | IGF1R, ME1, FABP4, TGFB1, TGFBR2, MYC, HMOX1, TLR2, TLR4, C1QB, CITED1, B2M, ACTR3, CD48, GSTM3, THBS1, MYD88, ANG, LOX, CASP1, KCNMA1, IRAK2, BCL10, LYN, GATM, LY96, SOCS3, MGP, PRKCQ, CD83, ADM, NME1, SLC25A36, CFL1, RIPK2, HSPB3, COL1A1, CCL2, ENPP1, COL3A1, NNAT, CDH1, COMT, EDEM1, MANFSLC11A1, DGKD, BCL2, TXNIP, MSH2, KLF10, MAP1B, CDH13, CDKN1A, EPS8, VCP, ARSA, ID3, AACS, PARP1, CD14 |
| GO:0070482~response to oxygen levels | HMOX1, TGFB1, IL18, CDKN1A, KCNMA1, ATP1B1, CCL2, PDPN, SOCS3, PRKCQ, ADM, ANG, BCL2, CASP1, THBS1, PLAU, ANGPTL4 |
| GO:0001501~skeletal system development | MYC, IGF1, IGFBP5, TGFB1, TGFBR2, OSTF1, COL1A1, COL12A1, COL5A2, COL3A1, SOX9, VDR, CTSK, COMP, BCL2, GPNMB, KLF10, ARID5B, MGP, WWTR1, AMBN, TULP3, ETS2, STC1, PLEKHA1 |
| GO:0042981~regulation of apoptosis | ANXA1, ANXA4, IGF1, IGF1R, TGFB1, MYC, HMOX1, TRAF4, TLR2, TLR4, VAV2, VAV1, WFS1, CRADDBTK, CASP5, MYD88, TIAM1, CASP1, KCNMA1, BCL10, SOCS3, NLRP1, NME1, F3, CFL1, RIPK3, AVEN, RIPK2, CTSB, BID, CCL2, CLU, CDH1, SFN, ITM2B, SOX9, VDR, BCL2, COMP, THBS1, ANGPTL4, TXNIP, ABR, MSH2, KLF10, LGALS1, WRN, PLEKHF1, CDH13, CDKN1A, CSRNP3, VCP, ID3, IKBKB |
| GO:0009743~response to carbohydrate stimulus | ME1, TGFB1, TGFBR2, THBS1, TXNIP, ACTR3, PRKCQ, LYN, NNAT, MAP1B |
| GO:0006631~fatty acid metabolic process  (GO:0008610~lipid biosynthetic process) | FABP4, LIPA, HADH, ACACB, ACOT8, PLA2G15, AACS, ELOVL1, ELOVL5, PRKAB1, PTGDS, PDPN, SYK, DECR1, ABHD5, TBXAS1 |
| GO:0048639~positive regulation of developmental growth | MYOD1, TGFBR2, MAP1B, HOPX |

^1^Gene ontology (GO) identification number and term of the biological process.

^2^Unique genes included in each pathway.
