# Supplementary material for: A transcriptome multi-tissue analysis identifies biological pathways and genes associated with variations in feed efficiency of growing pigs
Source: BMC Genomics. 2017 Mar 21;18:244. doi: 10.1186/s12864-017-3639-0 (PMC5361837; doi:10.1186/s12864-017-3639-0)
Supplement: Supplementary file 9 — Genes participating to relevant GO biological processes specifically in liver. (DOCX 22 kb) [file 12864_2017_3639_MOESM9_ESM.docx]

**Additional file 9** *Genes participating to relevant GO biological processes in liver as affected by selection for RFI*

| Biological processes^1^ | Genes^2^ |
| --- | --- |
| **Liver:** Overexpressed in low RFI pigs compared with high RFI pigs | |
| GO:0009100~glycoprotein metabolic process | PSEN2, ST3GAL1, ST3GAL5, STT3A, MAN1A2, ALG2, LIPC, POFUT1, HS2ST1 |
| GO:0043691~reverse cholesterol transport  (GO:0006869~lipid transport) | APOA1, APOAF, LCAT, LIPC, OSBPL3, C11ORF2 |
| GO:0007242~intracellular signaling cascade | GCK, APOA1, MAP4K4, GNAZ, GNA13, ADCY4, PTPLAD1, FGF13, TLR5, C1QTNF2, PLCD3, GUCY1A3, GLP2R, PAG1, ADAM9, RET, RINT1, RAB7L1, RGNEF, TANK, FARP2, ADRB1, PRLR, NTRK1, PSEN2, TGFBR3, CLEC7A, GRB14 |
| **Liver:** Under-expressed in low RFI pigs compared with high RFI pigs | |
| GO:0006952~defense response | IL10, IL10RB, C1QA, TNFAIP8L2, CFP, BCL3, MASP2, LY75, CEBPB, FGR, NCF1, HCK, SGMS1, CCL5, CHST1, SIGLEC1, LILRB5 |
| hsa00591:Linoleic acid metabolism | PLA2G4A, CYP3A4, CYP3A5, CYP2J2, CYP1A2 |
| GO:0042981~regulation of apoptosis | TRAF2, TRAF3, TNFSF13B, IL10, BCL3, TGFBR1, PIK3CG, IER3, NGEF, CEBPB, TBX5, SGMS1, TIMP3, MCF2L, NME2, PLA2G4A, CDKN2D, PRKRA, HSPB1 |
| GO:0033559~unsaturated fatty acid metabolic process | PLA2G4A, CYP2J2, ALOX5AP, MGST2 |
| GO:0006468~protein amino acid phosphorylation | TGFBR1, PIK3CG, PLK3, PRKRA, FGR, HCK, ADRBK2, ABI2, CASK, PBK, GUCY2C, CAMK2D, LMTK2, TSSK4, CSK, SIK3 |
| GO:0050778~positive regulation of immune response | TRAF2, TNFSF13B, C1QA, CFP, MASP2, CD247, SH2B2 |
| GO:0006959~humoral immune response | C1QA, CFP, BCL3PSMB10, MASP2, AIRE |

^1^Gene ontology (GO) identification number and term of the biological process.

^2^Unique genes included in each pathway.
