# Supplementary material for: A transcriptome multi-tissue analysis identifies biological pathways and genes associated with variations in feed efficiency of growing pigs
Source: BMC Genomics. 2017 Mar 21;18:244. doi: 10.1186/s12864-017-3639-0 (PMC5361837; doi:10.1186/s12864-017-3639-0)
Supplement: Supplementary file 10 — Genes participating to relevant GO biological processes specifically in adipose tissues. (DOCX 22 kb) [file 12864_2017_3639_MOESM10_ESM.docx]

**Additional file 10** *Genes participating to relevant GO biological processes in adipose tissues as affected by selection for RFI*

| Biological processes^1^ | Genes^2^ |
| --- | --- |
| **SCAT:** Overexpressed in low RFI pigs compared with high RFI pigs | |
| GO:0006796~phosphate metabolic process | GPD1, PDK4, SOD1, LIMK2, ALPK2, NUAK1, ERBB2, HUS1, MINK1, RPS6KA3, THTPA, MAPK7, CDK16, AKT3 |
| GO:0006006~glucose metabolic process | GPD1, PDK4, PC, ADIPOQ, NISCH |
| GO:0006091~generation of precursor metabolites and energy  (GO:0016042~lipid catabolic process)  (GO:0006635~fatty acid beta-oxidation) | PLIN1, ACADVL, ACAA1, ADIPOQ, SUCLA2, THTPA, TXNRD1, CAT, CYB5A, PLCD4, SRD5A2 |
| GO:0034599~cellular response to oxidative stress | CAT, SOD1, DUOX2, HMOX2 |
| **SCAT:** Under-expressed in low RFI pigs compared with high RFI pigs | |
| GO:0007517~muscle organ development | GATA6, SVIL, ELN, CHODL, SGCE, MYH10 |
| GO:0006796~phosphate metabolic process | TGFB3, VRK1, ALPK1, CDKL1, FXN, MTMR10, NTRK2, MATK, AATK |
| GO:0006952~defense response | CCL21, CCL8, CFD, COLEC12, ATRN, BECN1, BDKRB2 |
| **PRAT:** Under-expressed in low RFI pigs compared with high RFI pigs | |
| GO:0001568~blood vessel development | NRP2, HOXA3, HAND1, ZMIZ1, JAG1, EGF, GJA5, ANGPT2, CEACAM1, GJC1 |
| GO:0008202~steroid metabolic process  (GO:0008203~cholesterol metabolic process) | CYP7B1, CYP1B1, SREBF2, CEL, STARD5, TFCP2L1, SCARF1 |

^1^Gene ontology (GO) identification number and term of the biological process for subcutaneous (SCAT) or perirenal (PRAT) adipose tissues.

^2^Unique genes included in each pathway.
